# Correction: Digital inclusive finance penetration and household debt maturity structure: Empirical estimation based on China Family Panel Studies Data

**DOI:** 10.1371/journal.pone.0337229

**Published:** 2025-11-18

**Authors:** Jiayu Hou, Yingli Zhang

The first author, Jiayu Hou, is incorrectly noted as the corresponding author. The correct corresponding author is Yingli Zhang. Yingli Zhang’s email address is: yl-zhang@shou.edu.cn.
