# Supplementary material for: Digital inclusive finance penetration and household debt maturity structure: Empirical estimation based on China Family Panel Studies Data
Source: PLoS One. 2025 Apr 29;20(4):e0320080. doi: 10.1371/journal.pone.0320080 (PMC12040078; doi:10.1371/journal.pone.0320080)
Supplement: S1 Data — (ZIP) [file pone.0320080.s001.zip › Research Data/Digital Inclusive Financial Index.pdf]

北京大学数字普惠金融指数

（2011-2020 年）

北京大学数字金融研究中心课题组[①]

课题组顾问

黄益平　北京大学数字金融研究中心主任

李振华　蚂蚁集团研究院院长

课题组成员

郭峰、王靖一、王雪、程志云、李勇国、王芳

技术支持团队

陈前进、邓中华、傅志斌、郭亮、刘朋、刘晓辉、聂正军、王纪东、王强、袁胜雄、周卫林、郑明

2021 年 4 月

① 本指数是国家社会科学基金重大项目“数字普惠金融的创新、风险与监管研究”（课题号：18ZDA091）的阶段性成果，在指数编制和分析过程中，课题组得到了北京大学数字金融研究中心和蚂蚁集团各位领导和同事的大力支持和帮助，特此致谢。本指数不代表北京大学数字金融研究中心和蚂蚁集团的观点，所有问题由课题组负责。

目录

一、内容提要 1

二、数字普惠金融发展最新趋势 5

（一）数字普惠金融的增长情况 6
（二）数字普惠金融的地区差异 8
（三）数字普惠金融地区排行榜变化 13

三、数字普惠金融发展空间格局专题分析 16

（一）数字普惠金融南北差异 16
（二）数字普惠金融城乡差异 18

四、结论 23

参考文献 25

附录 1：省级数字普惠金融指数（2011-2020） 26

附录 2：数字普惠金融指标体系与指数计算方法 36

附录 3：使用数字普惠金融指数的代表性学术成果 43

附录 4：北京大学数字金融研究中心简介 45

一、内容提要

普惠金融（financial inclusion）可以定义为能有效和全方位地为社会所有阶层和群体提供服务的金融体系，其初衷意在强调通过金融基础设施的不断完善，提高金融服务的可得性，实现以较低成本向社会各界人士，尤其是欠发达地区和社会低收入者提供较为便捷的金融服务。这一概念最初被联合国用于“2005 年国际小额信贷年”的宣传中，后被联合国和世界银行大力推广。到 2014 年世界银行已在全球 70 多个国家和地区与公私合作伙伴联手开展普惠金融项目，全世界 50 多个国家和地区设立了改善普惠金融的目标（世界银行集团，2015）。2005 年后，普惠金融的概念引入中国，并得到中国政府的认可。2013 年 11 月，党的十八届三中全会通过《中共中央关于全面深化改革若干重大问题的决定》，正式提出发展普惠金融的理念。2015 年底，国务院发布《推进普惠金融发展规划（2016-2020）》，对普惠金融事业进行了更具体的部署，并在其中明确了普惠金融的定义：普惠金融是指立足机会平等要求和商业可持续原则，通过加大政策引导扶持、加强金融体系建设、健全金融基础设施，以可负担的成本为有金融服务需求的社会各阶层和群体提供适当的、有效的金融服务。

无论是在国际上还是在中国国内，普惠金融的概念、理论和实践都经历了一个逐步深化的过程：从最初重点关注银行物理网点和信贷服务的可获得性，到广泛覆盖支付、存款、贷款、保险、信用服务和证券等多种业务领域（焦瑾璞等，2015）。在实践层面，中国普惠金融实践已经从最初的公益性小额信贷逐步扩展为支付、信贷等多业务的综合金融服务，并由于网络和移动通讯等技术的广泛应用而得到长足发展。当前，中国普惠金融的实践与创新型数字金融显示出很强的关联性，以互联网科技企业提供金融服务为代表的新型数字金融业务，通过信息化技术及产品创新，降低金融服务产品的成本，扩大金融服务的覆盖范围，因此新型数字金融模式已经成为普惠金融的重要源动力和增长点。具体而言，从覆盖的区域来看，由于传统金融业务需要通过设置机构网点来提高覆盖面，但机构网点的高成本导致传统金融业务难以渗透到经济相对落后地区。而数字技术与金融服务的跨界融合克服了这种弊端，一些地区即便没有银行网点、ATM 等硬件设施，客户仍能通过电脑、手机等终端设备获得所需的金融服务。与传统金融机构将主要资源分布于人口、商业集中地区的状况相比，数字金融使得金融服务更直

接，客户覆盖面更广泛。从覆盖的社会群体来看，数字金融的产品创新降低了客户准入门槛，使得金融服务平民化趋势更加显现。与传统金融机构的排他性对比，数字金融可以满足那些通常难以享受到金融服务的中小微企业和低收入人群的需求，从而体现了普惠金融的应有之义。

过去数年，中国数字金融取得了长足发展，在全球都产生了很大影响力（黄益平和黄卓，2018），但却一直缺乏一个衡量其总体发展水平的指标体系。为此，北京大学数字金融研究中心和蚂蚁集团研究院的研究团队自 2016 年开始，利用蚂蚁集团关于数字普惠金融的海量数据，编制了一套“北京大学数字普惠金融指数”（The Peking University Digital Financial Inclusion Index of China，PKU_DFIIC），并在 2019 年对指数进行了更新，本报告是该指数的第三次更新（郭峰等，2016，2019，2020）。在现有文献和国际组织提出的传统普惠金融指标基础上，结合数字金融服务新形势、新特征与数据的可得性和可靠性，课题组从数字金融覆盖广度、数字金融使用深度和普惠金融数字化程度等 3 个维度来构建数字普惠金融指标体系。目前数字普惠金融指数一共包含上述 3 个维度，33 个具体指标。基于上述指标体系和类似文献中常用的指数编制方法“层次分析法”，课题组最终编制了中国内地 31 个省（直辖市、自治区）、337 个地级以上城市（地区、自治州、盟等），以及约 2800 个县（县级市、旗、市辖区等）三个层级的“北京大学数字普惠金融指数”。本次更新后，本指数包括了 2011-2020 年的省级和城市级指数，2014-2020 年的县域指数。指数同时具有纵向和横向上的可比性。在总指数基础上，课题组还提供了数字金融覆盖广度指数、数字金融使用深度指数和普惠金融数字化程度指数，以及数字金融使用深度指数下属的支付、保险、货币基金、信用服务、投资、信贷等分类指数。

编制这套指数的目的是在不泄露金融消费者个人隐私和金融机构商业机密的前提下，为各界提供一套反映数字普惠金融发展现状和演变趋势的工具性数据。本报告旨在以指数编制的形式对中国的数字普惠金融实践进行定量刻画。这一努力至少具有三方面的重要意义：第一，从理论上看，本指数将为国内创新性普惠金融研究以及统计指标体系设计提供重要参考。国内现有关于普惠金融的研究主要集中于从传统金融服务的角度来研究普惠金融的概念、意义、指标构造和作用等，尚无一套从创新性数字金融角度来科学、全面地概括中国现阶段数字普惠金

融的理论和指标体系。本报告通过梳理目前国内外关于普惠金融指标体系和指数的研究，并结合现阶段国内创新性数字金融快速发展的实际情况，构建数字普惠金融指数体系，推动普惠金融研究的深化。第二，从实践意义上看，基于构建的指标体系，本报告编制各省份、各城市以及县域级的数字普惠金融指数，反映中国创新性数字金融趋势下数字普惠金融发展程度和地区均衡程度。这将有助于政策制定者和从业者更好地了解中国数字普惠金融的发展现状，识别数字普惠金融发展面临的瓶颈与障碍，为相关政策的制定提供依据，促进数字普惠金融健康可持续发展。第三，从实际效果上看，指数自 2016 年第一次发布，2019 年、2021 年两次更新，已经成为在中国金融科技、普惠金融的研究者中颇具影响力的数据产品，一方面许多学者研究成果以不同角度分析了数字普惠金融的发展成因与影响，另一方面，愈发蓬勃的发表、工作论文与公开报告吸引越来越多的学者与博士生加入到数字普惠金融的研究之中。

在本次报告中，我们基于该指数的最新数值（2011-2020），对中国数字普惠金融的最新发展趋势进行了分析，并重点讨论了数字普惠金融在南北区域和城乡之间的问题，得到如下几个主要结论：第一，经历了早期的高速发展后，数字普惠金融发展速度近几年有所放缓，但仍然维持了一个可观的增速，特别是新冠肺炎疫情之后，数字普惠金融服务依然增长，近十年来数字普惠金融的实践初步证明了这种模式的可行性、商业可持续性和可复制性。第二，与前几年的指数相比，最近几年指数增长的驱动力发生了非常明显的变化，数字金融使用深度的增长已经成为数字普惠金融指数增长的重要驱动力，这与第一期 2011-2015 年的指数形成鲜明对照，证明中国的数字普惠金融事业已经走过了粗放式的圈地时代，进入了深度拓展的新阶段。第三，前几年数字普惠金融区域间差异收敛速度明显，但近两年有所放缓，未来差异能否进一步缩小，主要取决于使用深度的地区差异能否进一步缩小。第四，中国北方和南方的数字普惠金融发展水平的差距由前两年的快速缩小，转为近两年的速度放缓，北方地区和南方地区的数字普惠金融发展差异，主要体现在使用深度上。第五，在城乡层面，总体而言，城市化水平与数字金融发展正相关，但具体到比较核心、发展水平已经比较高的都市区内部，其人口集中度与数字金融发展是负相关关系。

本报告剩余部分的结构安排如下：第二部分对数字普惠金融指数反映出的

中国数字普惠金融发展的最新趋势进行分析，同时也重点讨论了2020年新冠肺炎疫情爆发后，中国数字普惠金融指数的特别变化；第三部分侧重于南北和城乡的视角，对数字普惠金融反映出的中国区域经济格局及其变化进行了分析；第四部分简要总结了本报告得到的核心结论。同时在报告最后，我们也整理了省级数字普惠金融指数的数值、数字普惠金融指数的指标体系和指数编制方法以及使用本指数发表的一些代表性学术成果，方便大家参考。

最后需要说明的是，我们在本报告中所分析的只是数字普惠金融指数特征的一些直观特征，这套指数更重要的价值在于与其他经济社会特征匹配起来进行更深入的分析。本指数最早于 2016 年发布第一期，2019 年和 2021 年分别发布第二期和第三期，截至目前已有上千人向课题组索取过该指数。使用本数据开展的研究，已经基本实现了对《经济研究》、《经济学》（季刊）、《管理世界》、《金融研究》、《财贸经济》等中文权威经济学期刊的全覆盖（详见报告附录 3）。因此，我们欢迎各界人士使用这套指数，指数全部数据可向课题组免费索取：pku_dfiic@163.com。如有使用本数据，请注明所用数据为“北京大学数字普惠金融指数”，同时烦请按照以下文献引用方式引用我们的成果：**郭峰、王靖一、王芳、孔涛、张勋、程志云，《测度中国数字普惠金融发展：指数编制与空间特征》，2020 年第 19 卷第 4 期，第 1401-1418 页。**

二、数字普惠金融发展最新趋势

在这套指数中，我们编制了全国内地 31 个省（直辖市、自治区，简称“省”）、337 个地级以上城市（地区、自治州、盟等，简称“城市”），以及约 2800 个县（县级市、旗、市辖区等，简称“县域”）三个层级的数字普惠金融指数②，省级和城市级指数时间跨度为 2011-2020 年，县域指数时间跨度为 2014-2020 年。在总指数基础上，我们还从不同维度编制了数字普惠金融的覆盖广度、使用深度和数字化程度指数，以及支付、保险、货币基金、信用服务、投资、信贷等分类指数。2011 年-2020 年 31 个省的数字普惠金融指数如表 1 所示③。

表1：2011年-2020年省级数字普惠金融指数

省份	2011	2012	2013	2014	2015	2016	2017	2018	2019	2020
北京市	79.41	150.65	215.62	235.36	276.38	286.37	329.94	368.54	399.00	417.88
天津市	60.58	122.96	175.26	200.16	237.53	245.84	284.03	316.88	344.11	361.46
河北省	32.42	89.32	144.98	160.76	199.53	214.36	258.17	282.77	305.06	322.70
山西省	33.41	92.98	144.22	167.66	206.30	224.81	259.95	283.65	308.73	325.73
内蒙古	28.89	91.68	146.59	172.56	214.55	229.93	258.50	271.57	293.89	309.39
辽宁省	43.29	103.53	160.07	187.61	226.40	231.41	267.18	290.95	311.01	326.29
吉林省	24.51	87.23	138.36	165.62	208.20	217.07	254.76	276.08	292.77	308.26
黑龙江省	33.58	87.91	141.40	167.80	209.93	221.89	256.78	274.73	292.87	306.08
上海市	80.19	150.77	222.14	239.53	278.11	282.22	336.65	377.73	410.28	431.93
江苏省	62.08	122.03	180.98	204.16	244.01	253.75	297.69	334.02	361.93	381.61
浙江省	77.39	146.35	205.77	224.45	264.85	268.10	318.05	357.45	387.49	406.88
安徽省	33.07	96.63	150.83	180.59	211.28	228.78	271.60	303.83	330.29	350.16
福建省	61.76	123.21	183.10	202.59	245.21	252.67	299.28	334.44	360.51	380.13
江西省	29.74	91.93	146.13	175.69	208.35	223.76	267.17	296.23	319.13	340.61
山东省	38.55	100.35	159.30	181.88	220.66	232.57	272.06	301.13	327.36	347.81
河南省	28.40	83.68	142.08	166.65	205.34	223.12	266.92	295.76	322.12	340.81
湖北省	39.82	101.42	164.76	190.14	226.75	239.86	285.28	319.48	344.40	358.64
湖南省	32.68	93.71	147.71	167.27	206.38	217.69	261.12	286.81	310.85	332.03
广东省	69.48	127.06	184.78	201.53	240.95	248.00	296.17	331.92	360.61	379.53
广西	33.89	89.35	141.46	166.12	207.23	223.32	261.94	289.25	309.91	325.17
海南省	45.56	102.94	158.26	179.62	230.33	231.56	275.64	309.72	328.75	344.05
重庆市	41.89	100.02	159.86	184.71	221.84	233.89	276.31	301.53	325.47	344.76
四川省	40.16	100.13	153.04	173.82	215.48	225.41	267.80	294.30	317.11	334.82

② 2014-2015 年的县域指数仅包括县和县级市等地区，不包括市辖区，2016-2020 年县域指数则包含市辖区。本次指数更新基于之前的编制方法，详见附录。报告正文仅讨论该指数反映出的中国数字普惠金融发展的最新趋势，基于前两期指数对数字普惠金融的更全面分析也可以参阅我们课题组之前的报告和论文（郭峰等，2016，2019；2020；王靖一等，2019），以及附录 3 中基于该指数和其他外部数据匹配后进行研究得到的更丰富的研究发现。
③ 分类指数详见附件，城市和县域指数可通过联系课题组获取（pku_dfiic@163.com）获取。

贵州省	18.47	75.87	121.22	154.62	193.29	209.45	251.46	276.91	293.51	307.94
云南省	24.91	84.43	137.90	164.05	203.76	217.34	256.27	285.79	303.46	318.48
西藏	16.22	68.53	115.10	143.91	186.38	204.73	245.57	274.33	293.79	310.53
陕西省	40.96	98.24	148.37	178.73	216.12	229.37	266.85	295.95	322.89	342.04
甘肃省	18.84	76.29	128.39	159.76	199.78	204.11	243.78	266.82	289.14	305.50
青海省	18.33	61.47	118.01	145.93	195.15	200.38	240.20	263.12	282.65	298.23
宁夏	31.31	87.13	136.74	165.26	214.70	212.36	255.59	272.92	292.31	310.02
新疆	20.34	82.45	143.40	163.67	205.49	208.72	248.69	271.84	294.34	308.35

数据来源：北京大学数字普惠金融指数

（一）数字普惠金融的增长情况

以省级数据为例，如表 1 和图 1 所示，中国的数字普惠金融业务在 2011-2020 年间实现了跨越式发展，2011 年各省数字普惠金融指数的中位值为 33.6，到 2015 年增长到 214.6，2020 年进一步增长到 334.8。2020 年省级数字普惠金融指数的中位值是 2011 年的 10 倍，指数值平均每年增长 29.1%，由上可以看出中国数字普惠金融的快速发展趋势。从增速来看，最近几年数字普惠金融指数增速有所放缓，一定程度上表明随着数字金融市场的发展越来越成熟，该行业开始由高速增长阶段向常态增长过渡。2020 年，我国经济社会各方面均受到新冠疫情的严重冲击，全年经济增速较往年显著下降，但数字普惠金融指数仍然比 2019 年增长 5.6%。而且，进一步的分析也显示，即便是疫情最严重的武汉市和湖北省，虽然曾经封城数十天，数字普惠金融指数仍然维持正的增长，显示了数字金融在疫情时代的独特优势和强大韧性。

实际上，中国发达的数字金融和数字经济，在中国抗击防疫，包括缓解疫情对经济的冲击中发挥了非常重要的作用（郭峰，2021）。例如，最早从 2020 年 2 月起，杭州市就在全国最早启用了“健康码”防控措施[4]。根据 Xiao（2020）的研究，健康码对中国在做好防疫工作的同时推动经济活力的恢复，发挥了非常重要的作用，具体而言，该研究估算，“健康码”背后的大数据技术为中国疫情期间 GDP 增长做出了 0.5-0.75 个百分点的贡献。2020 年 2 月，我们课题组也基于本套数字普惠金融指数的底层数据和支付宝“码商”数据，研究了数字金融对于缓解疫情冲击的贡献。疫情对线下微型商户造成了严重的短期冲击，但全国总体而言，基于数字技术精准释放的信贷每增长 1%，疫情的冲击幅度就下降 2.57%。

[4] 浙江省市场监督管理局，《传染病防控人员健康码管理规范》，浙江省地方标准，DB33/T 2242-2020，2020 年 2 月 29 日。

为了对金融科技的该影响有更直观的感受，我们比较了基于数字技术精准发放的信贷发展水平较好的代表性城市杭州市与全国平均发展水平之间的发展差异，据此可以得到如下结论：如果一个地区基于数字技术精准发放的信贷发展水平可以从全国平均水平上升到杭州市的发展水平，则可以使得疫情冲击幅度下降约 51%，即疫情的冲击减弱约一半（王靖一等，2020）。

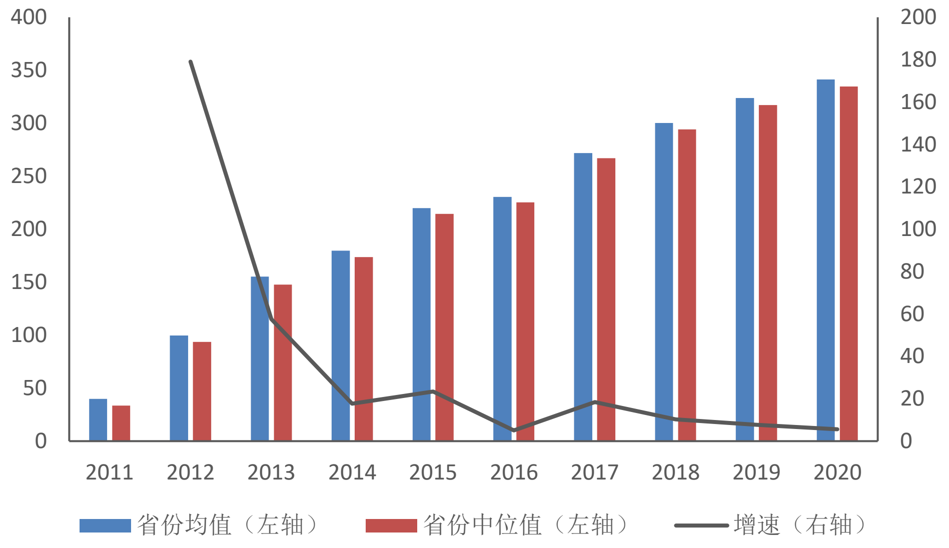

图1：2011-2019年省级数字普惠金融指数的均值、中位值和增速（%）

数据来源：北京大学数字普惠金融指数

从分指数来看，在 2016 年对 2011-2015 年的第一期指数的分析中，我们曾发现 2011-2015 年当中，数字普惠金融的数字化程度增长最快，数字普惠金融的覆盖广度次之，使用深度增长最慢。但在对 2016-2018 年第二期指数的分析中我们又发现，2016 年后指数增长情况发生了一些变动，数字金融使用深度指数增长有超过数字金融覆盖广度指数的趋势。最新的数据也进一步确认了这一趋势：2020 年数字金融使用深度指数较上年增长 7.3%，快于数字金融覆盖广度指数和数字技术支持程度指数 7.2%和-0.1%的增速。2016-2020 年 5 年间，有 4 年使用深度指数增速超过了覆盖广度指数，数字金融使用深度已经是数字普惠金融指数增长的重要驱动力⑤。对此，图 2 和图 3 中也有非常直观的展示。随着数字普惠金融的覆盖广度和数字支持程度达到一定程度，其进一步拓展的空间有限，目前各分指数近几年的增长趋势显示，未来数字金融的发展，将主要依赖于使用深度的增长空间。

⑤ 通过分析 2018 年使用深度数据，其增速下降的原因主要是在政策限制等因素影响下，货币基金指数和投资指数有所下降，其他几个使用深度指标依然保持增长趋势。

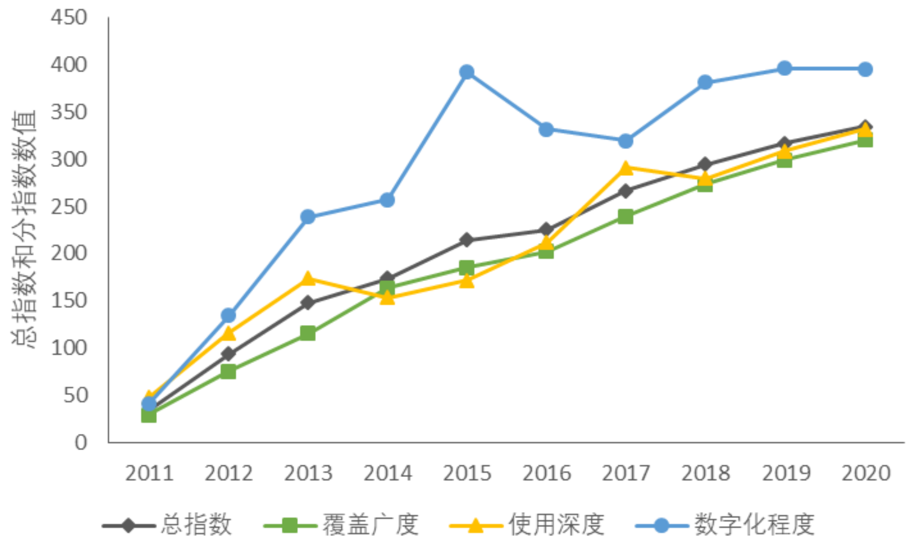

图2：2011-2020年数字普惠金融指数及分指数的省级中位值

数据来源：北京大学数字普惠金融指数

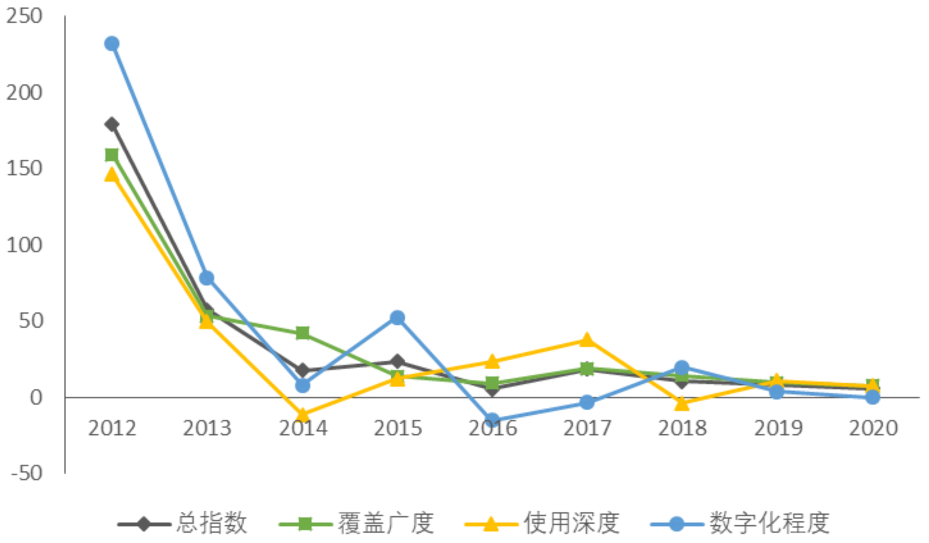

图3：2011-2020年数字普惠金融指数及分指数的省级中位值增长率

数据来源：北京大学数字普惠金融指数

（二）数字普惠金融的地区差异

在之前两期报告中，我们都重点讨论数字普惠金融指数反映出的地区间数字普惠金融发展差距问题，本次数据更新亦延续此前的分析。从图 4 中可以看出，上海市、北京市以及数字经济活跃的浙江省，数字普惠金融指数明显比其他几个地区更高，处于第一梯队；而指数在西藏之后的省份均是地处西部地区或东北地区的省份，数字普惠金融发展水平明显较低；其他东部和中部省份则处于上述两

个梯队之间。

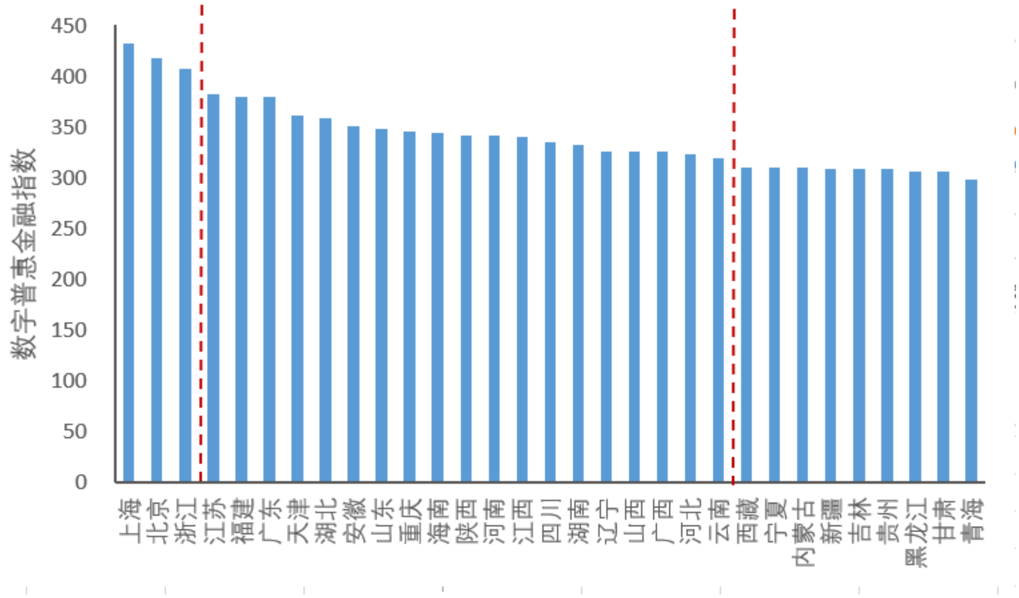

图4：2020年各省数字普惠金融指数分布

数据来源：北京大学数字普惠金融指数

就具体分指数的地区差异而言，如图 5 所示，普惠金融的数字支持程度的地区差距最小、数字金融覆盖广度次之，数字金融使用深度地区差异最大。具体而言，数字金融的覆盖广度、使用深度和数字支持程度 2020 年指数最高的地区与最低的地区之比分别为 1.36、1.89 和 1.24。虽然相较于前几年，数字金融使用深度地区间差异已经大幅缩小，但仍然驱动了数字普惠金融指数的地区差距。在使用深度上，落后地区与发达地区相比，还有一定的差距。而在具体业态方面，互联网投资的地区差异明显高于其他几个业态。这跟数字金融的特性有很大关系，让更多的人接触、使用上数字金融服务，是相对容易的一件事，但促进既有用户对各类数字金融产品的广泛使用，以及提升用户对数字金融服务的依赖度，尚有较大的增长空间。

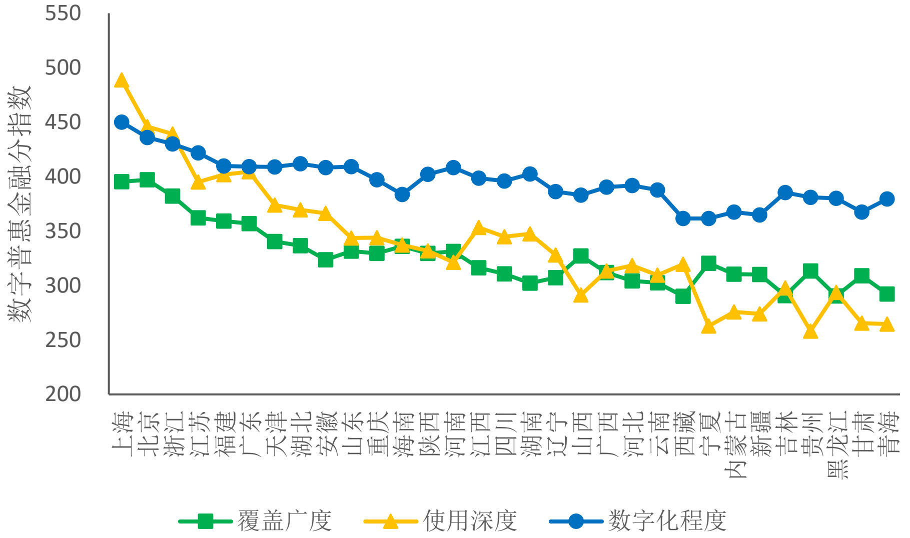

图5：2020年各省数字普惠金融分类指数分布

数据来源：北京大学数字普惠金融指数

另一个值得关心的问题是，地区之间数字普惠金融发展水平差距是否在逐步缩小？图 6 给出了城市层面的数字普惠金融指数排序：2011 年、2015 年和 2020 年的梯队分类标准以当年指数最高的城市指数值为基准，将排序在基准值 80%范围内的城市列为第一梯队，在图中标记为红色；70%-80%范围内为第二梯队，在图中标记为橙色；60%-70%为第三梯队，在图中标记为黄色；60%之后的城市列为第四梯队，在图中标记为绿色。从图 6 中我们可以发现，在 2011 年，城市之间发展存在较大的差距，第一梯队集中在上海邻近地区与大城市，且二三梯队十分单薄，大部分地区处于第四梯队；在 2015 年，第一梯队扩展至东南沿海与区域级重点城市，同时二三梯队发展壮大；而发展到 2020 年，绝大部分城市处于一二梯队，即绝大多数城市的数字普惠金融指数都在当年最高地区的 70%以内，地区之间的差距进一步缩小。不过，对比 2020 年和 2018 年的数据，也可以发现，在 2020 年处于第三、第四梯队的城市数量（74 个）比 2018 年的数量（63 个）还要多，这表明近期数字普惠金融的地区差异缩小的趋势可能有所放缓。

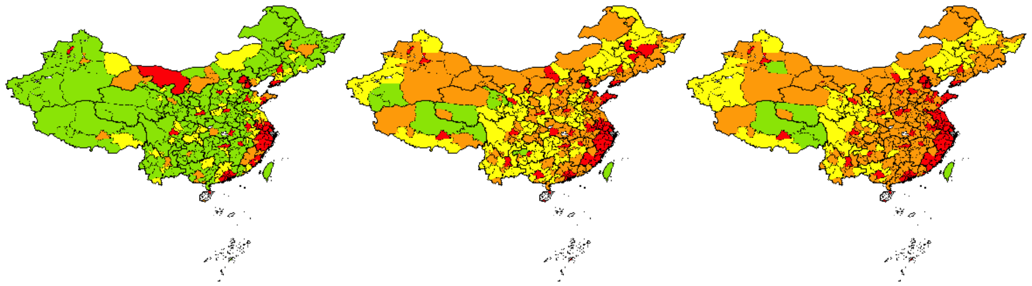

图6：2011、2015和2020年城市数字普惠金融总指数相对排序

数据来源：北京大学数字普惠金融指数

注：台港澳地区和部分省直辖县市缺少数据，因此为白色。

2019 年 9 月，我们曾撰写研究报告《数字经济助力中国东西部经济平衡发展》（王靖一等，2019），以地理经济学当中著名的“胡焕庸线”为东西部地区的划分标准，计算了数字金融触达性、覆盖广度等层面的东西部地区差异，发现这种差异有明显的下降趋势：数字经济跨越“胡焕庸线”，即以移动支付为代表的数字金融，为西部偏远地区的居民接触、使用先进的数字金融服务创造了条件，进而为中国区域经济的平衡发展创造了更多机遇。同时，我们基于数字普惠金融发表在《经济学》（季刊）上的论文（郭峰等，2020）对于使用深度在胡焕庸线两侧表现出的发展趋势也进行了分析。总指数的结论相对而言较不明晰，如果我们将覆盖广度与使用深度分别绘图，结果就相对更为直观。

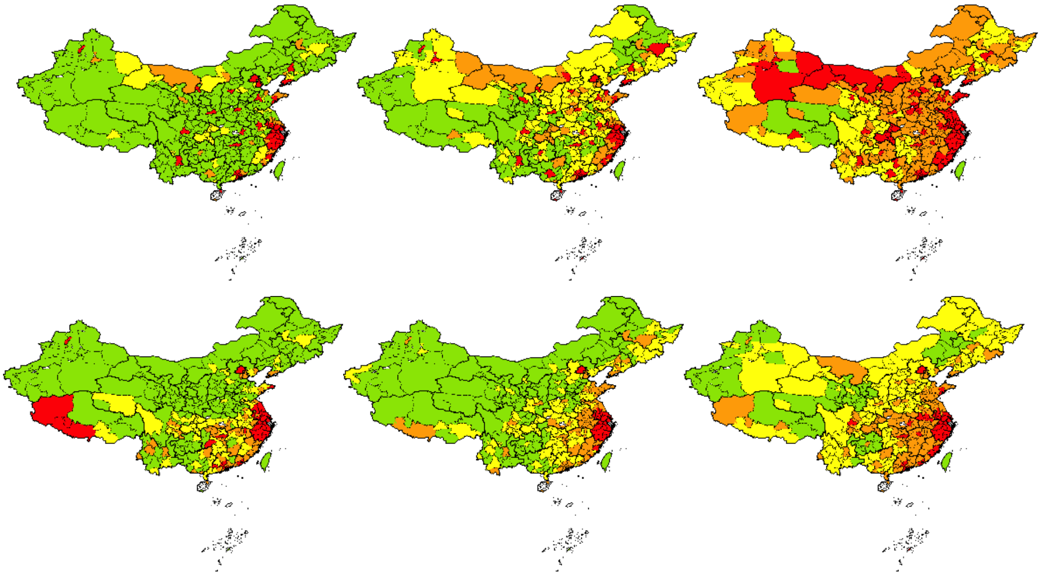

图7：2011、2015和2020年城市数字普惠金融覆盖广度（上列）使用深度（下列）相对排序

数据来源：北京大学数字普惠金融指数

注：台港澳地区和部分省直辖县市缺少数据，因此为白色。

如图 7 所示，数字金融覆盖广度在 2011-2020 年间保持了跨越胡焕庸线发展的趋势，但就使用深度而言，优势发展地区仍在东南集中。从图中我们可以看出深度与广度发展的一个重要区别是，广度的增长具有一定的跨越性，即部分中西部地区在相对水平与绝对水平上都实现了跨越式发展，在后期实现了对东南地区

的的大幅追赶，而深度的发展则是渐进式、绝对水平意义上的：虽然相较而言东南地区仍然具有明显的优势，中西部地区依旧相对落后，但绝对水平上，中西部地区与东部地区的差距仍在缩小。我们认为一个可能的原因是，覆盖广度衡量的是一种机会上的公平，即是否能够获得相关技术与服务的支持，而使用深度则体现着结果上的均衡，即最终发展情况位于何种水平，该结果取决于支持数字金融服务在当地发展的各种软硬环境。数字技术由于其脱离地理空间束缚、边际成本近乎为零的特点，在分布上促进了落后地区、人口稀疏地区的发展，让不同地区的居民可以共享普惠结果。而数字金融的本质仍是金融，金融服务的发展仍不能脱离经济活动生产生活而存在，由于集聚效应和网络效应，东部人口集中地区的发展水平、使用活跃程度仍将保持优势。数字金融的发展利用技术达到了获取服务的机会公平，而其服务实体的金融本质依然遵从相对集中的发展结果。

为了更严谨地论证地区数字普惠金融发展差距的时间趋势，我们也借助经济学中关于地区经济收敛性的论证方法进行讨论（Barro and Sala-i-Martin，1992；Sala-i-Martin，1996）。相关文献中，经济收敛的主要验证方法是σ收敛模型和β收敛模型。我们这里仅讨论其σ收敛趋势。

σ收敛是针对存量水平的刻画，反映的是地区数字普惠金融偏离整体平均水平的差异以及这种差异的动态过程，即如果这种差异越来越小，则可以认为地区数字普惠金融存在收敛性。具体而言，σ收敛模型可以定义为：

$$\sigma_t = \sqrt{\frac{1}{n}\sum_{i=1}^{n}(lnindex_{it} - \frac{1}{n}\sum_{i=1}^{n} lnindex_{it})^2} \qquad (3)$$

其中，i 代表地区（省、地市和县域等），n代表地区数量，t 代表年份，$lnindex_{it}$代表 t 年 i 地区的数字普惠金融指数对数值，σ_t代表 t 年数字普惠金融指数的σ收敛检验系数。如果$\sigma_{t+1} < \sigma_t$则可以认为*t+1*年的数字普惠金融较 t 年更趋收敛。

在图8当中，我们分别汇报了2011-2020年省级和城市级数字普惠金融指数的σ收敛系数，从中可以看出，中国地区数字普惠金融在前几年的确有非常明显的收敛趋势，且收敛速度较快。具体来看，中国省级和城市级数字普惠金融指数的σ收敛系数分别从2011年的0.44和0.34下降到2017年的0.08和0.09。但最近几年，地区收敛速度有所放缓。为了考察近两年地区收敛速度有所放缓的具体原因，我们在图9当中也汇出了几个分指数的收敛系数，从中可以看出数字金融覆盖广度和普惠金融数字支持程度两个分指数的收敛系数在最近几年维持下降趋势，但是

数字金融使用深度指数收敛系数则有所反弹，这是数字普惠金融地区间收敛速度放缓的主要原因。在指数发展高度依赖于触达性、覆盖广度时，数字金融的地区间差异收敛较快，但当数字金融发展进入使用深度驱动的新阶段时，我们发现地区的使用深度差异依然存在较大的弥合空间，很大程度上可以解释地区间总指数的收敛速度为什么会逐步放缓。

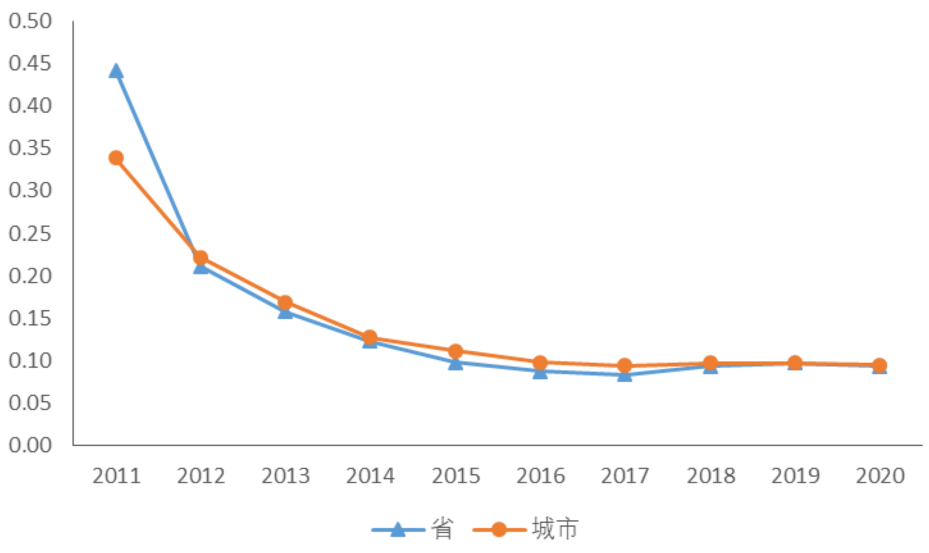

图 8:2011-2020 年省级和城市级数字普惠金融σ收敛系数

数据来源：北京大学数字普惠金融指数

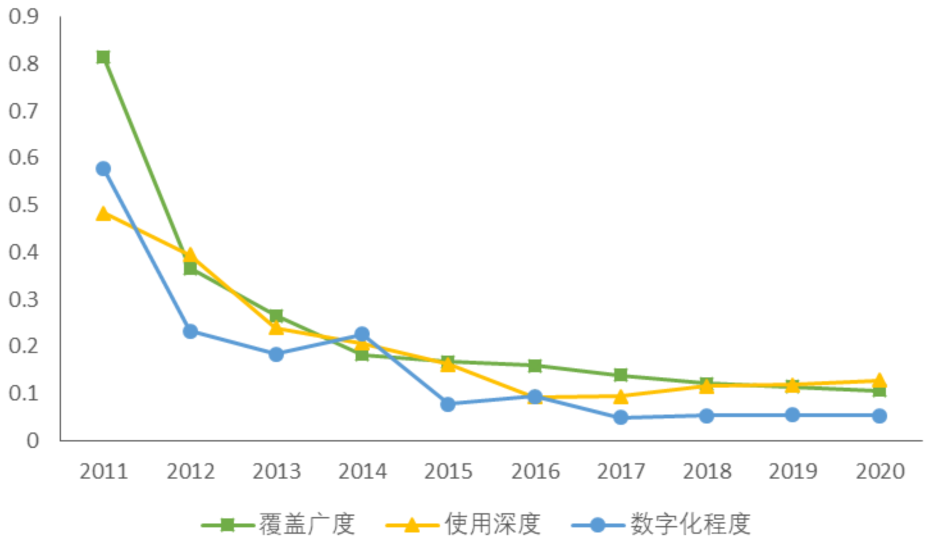

图 9:2011-2020 年城市级数字普惠金融分指数σ收敛系数

数据来源：北京大学数字普惠金融指数

（三）数字普惠金融地区排行榜变化

就 2020 年各省份的数字普惠金融的排行榜而言，可以发现该榜单与 2018 年相比，大多数地区排名变化不大，例如 31 个省（直辖市、自治区）中，有 17 个省排名不变或者仅上升（下降）1 位，说明了本指数的稳定性。不过也有个别地区在 2020 年排名变化稍大，内蒙古自治区排名较 2018 年上升 4 位，而贵州省和黑龙江省排名则分布下降 5 位和 4 位，是排名上升（下降）较多的几个省份。如果把时间窗口拉长，可以发现不同地区数字普惠金融指数排名还是有较大的变化。从表 2 中可以看出，黑龙江省、吉林省和辽宁省这三个东北省份的 2020 年指数较 2015 和 2011 年下降幅度较大。而地处中部地区的安徽、江西、河南等省份，排名则上升较多。

表2：2020年省份总指数排名及变化情况

省份名称	2020 年指数	排名	较 2018 年变化	较 2015 年变化	较 2011 年变化
上海	431.93	1	0	0	0
北京	417.88	2	0	0	0
浙江	406.88	3	0	0	0
江苏	381.61	4	1	1	1
福建	380.13	5	-1	-1	1
广东	379.53	6	0	0	-2
天津	361.46	7	1	0	0
湖北	358.64	8	-1	1	5
安徽	350.16	9	1	8	9
山东	347.81	10	2	2	4
重庆	344.76	11	0	0	-1
海南	344.05	12	-3	-4	-4
陕西	342.04	13	1	0	-2
河南	340.81	14	1	11	10
江西	340.61	15	-2	4	7
四川	334.82	16	0	-2	-4
湖南	332.03	17	2	5	2
辽宁	326.29	18	-1	-8	-9
山西	325.73	19	2	4	-2
广西	325.17	20	-2	1	-5
河北	322.70	21	1	7	-1
云南	318.48	22	-2	4	3
西藏	310.53	23	3	8	8
宁夏	310.02	24	3	-9	-3
内蒙古	309.39	25	4	-9	-2
新疆	308.35	26	2	-2	1
吉林	308.26	27	-3	-7	-1
贵州	307.94	28	-5	2	1

黑龙江	306.08	29	-4	-11	-13
甘肃	305.50	30	0	-3	-2
青海	298.23	31	0	-2	-1

数据来源：北京大学数字普惠金融指数

在城市层面，从表 3 当中可以看出，排名靠前的城市基本上都属于东部沿海省份，特别是江浙沪地区，扎堆现象明显。排名变化上，2020 年排名前 10 的城市和 2018 年相比，变化不是很明显。不过，如果跟 2011 年相比，有些城市会显示出明显的排名变化，比如深圳市和广州市的 2020 年排名，比 2011 年要提高很多，当然这种提升并不是近年才发生的，因为这两个城市的 2020 年排名，较 2015 年并没有太大变化。

表3：2020年城市排名前10及变化情况

城市名称	2020 年指数	排名	较 2018 年变化	较 2015 年变化	较 2011 年变化
杭州市	334.48	1	0	0	0
上海市	320.79	2	0	0	1
深圳市	319.24	3	0	1	18
厦门市	314.27	4	2	1	1
南京市	313.90	5	-1	1	-1
北京市	311.96	6	-1	-3	1
广州市	310.34	7	0	0	17
苏州市	309.80	8	0	6	2
金华市	307.33	9	3	3	-3
常州市	304.52	10	0	5	5

数据来源：北京大学数字普惠金融指数

不过，上述只针对排名靠前的城市的分析，很难观察出中国数字普惠金融在不同地区此消彼长的情况。在图 10 中，我们绘出了 2020 年全部城市排名中相对于 2018、2015，以及 2015 年排名相对于 2011 上升的城市（红色）和相对下降的城市（绿色）。其中第一幅图是 2020 年相对于 2018 年的排名变化情况，从中可以看出，在较短时间内，排名变化的空间格局是比较零碎的，不同区域均有上升和下降的城市，唯一连片上升的地区大致处于陕西-四川-云南一线，这些地区处于中国中西部交会地区。而 2020 年排名相对于 2015（第二幅图），以及 2015 年相较于 2011 年（第三幅图）就具有明显的空间格局：西南方向城市排名上升的数量明显多于北方，特别是东北方向的城市。

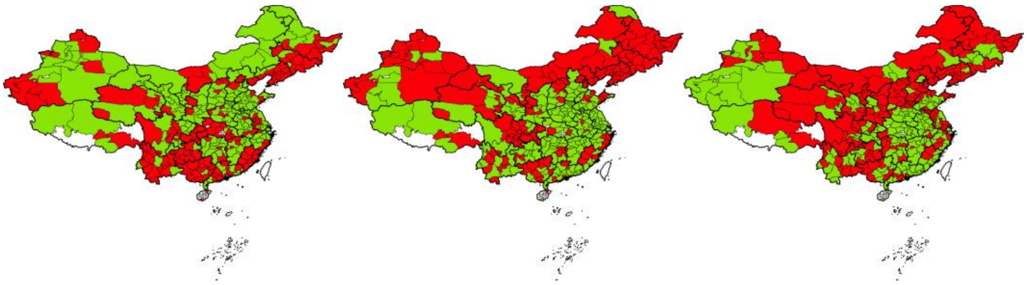

图10：城市级数字普惠金融指数排名变化

数据来源：北京大学数字普惠金融指数

注：左图为 2020vs2018 的排名变化；中图为 2020vs2015 的排名变化，右图为 2015vs2011 的排名变化。

三、数字普惠金融发展空间格局专题分析

在 2019 年的报告中，我们重点讨论了中国东西部地区数字普惠金融发展差距的变化趋势，得到的结论是中国东西部地区数字普惠金融发展差距在缩小，数字金融为西部地区企业和居民，克服地理条件限制，共享经济发展红利创造了条件。我们在本部分从南北和城乡的视角，对数字普惠金融发展的地区差异进行专题分析。

（一）数字普惠金融南北差异

中国南方和北方的经济关系，在上千年的历史上都是一个重要的话题，在数字金融飞速发展的近些年，是否呈现出新趋势？我们在本小节对此进行简要的分析。

关于南北分界线，仿照传统做法，我们也以秦岭-淮河为界，在地级市一级上将中国划分为南方和北方。我们首先简单比较了中国北方城市数字普惠金融指数均值与中国南方城市均值之比的变化趋势，如图 11 所示。从图 11 中可以得到如下几个结论：第一，中国南北数字普惠金融发展差距并不算太大，北方数字普惠金融发展水平略低于中国南方地区；第二，中国北方地区数字普惠金融总体上有追赶南方地区的趋势，特别是在 2011-2014 年之间，追赶速度很快，北方城市数字普惠金融指数均值由南方城市的 88%上升至 95%左右；第三，自 2018 年以来，中国南北数字普惠金融发展差距又有拉大的趋势，但数据时限较短，且差距拉大的幅度较小，所以趋势性变化不明显，有待进一步观察。

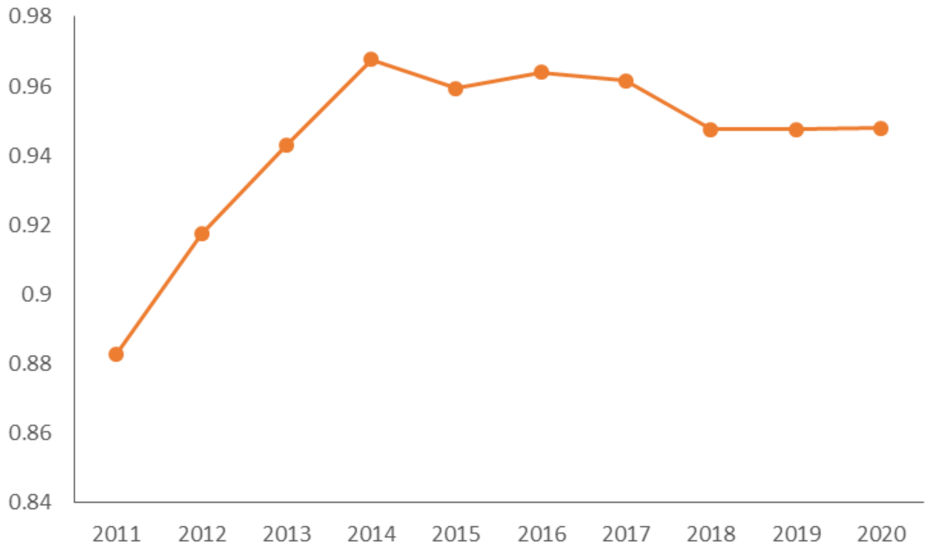

图 11：中国南北城市数字普惠金融指数差异变化趋势（北方均值/南方均值）

数据来源：北京大学数字普惠金融指数

图 12 展示了数字金融覆盖广度、数字金融使用深度和普惠金融数字化程度三个分指数南北之间差距的变化趋势，从中可以得到一些更丰富的结论：北方城市的数字化程度由领先于南方城市，近几年转而逐渐落后于南方城市，这是数字普惠金融总指数几年来南北差距略微拉大的主要原因。为深入探究北方地区数字化指数下降的原因，我们也对其分指数变化趋势进行了简要的梳理。结果发现，在数字化指数的四个分指数中，下降最明显的是实惠化和信用化，北方地区小微企业的融资利率环境相比于南方地区在变差（实惠化），以及信用分的使用场景有所下降，这说明数字金融的发展需要其他金融和硬件基础设施的相应配套才能有更大的发展空间。再看数字金融的使用深度，北方城市起初明显落后于南方城市，但随后几年迅速追赶，这是前几年北方城市数字普惠金融总指数能够追赶南方城市的主要因素，但总体而言，数字金融使用深度上的南北差距仍然是几个分指数中差距最大的一个。未来，北方地区数字普惠金融发展水平能否继续追赶南方地区，主要还是要看数字金融使用深度上能否缩小差距，这一点跟前文得到的结论是高度契合的。

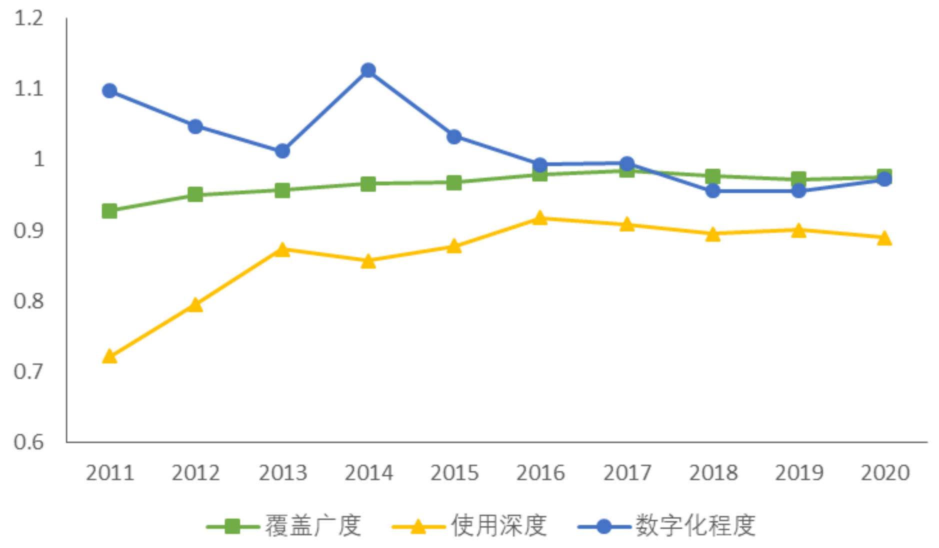

图 12：数字普惠金融分指数南北差异变化趋势（北方均值/南方均值）

数据来源：北京大学数字普惠金融指数

（二）数字普惠金融城乡差异

大规模的城市化是中国过去二十年非常突出的特征，也将仍然是未来一段时间内中国经济增长的重要源动力。因此我们在本节分析城乡分布与人口集中程度与数字普惠金融发展水平之间的关系。在之前的指数报告和学术论文中，我们分析了胡焕庸线两侧数字金融发展的异同关系（王靖一等，2019；郭峰等，2020），而胡焕庸线本质上反应了人口分布的集中度问题，故而本次我们实际上相当于在更小的空间颗粒度上，对人口集中度与数字金融发展之间的关系进行了更细致深入的分析。

为了科学精准地衡量城市化水平，特别是更小颗粒度空间上的人口空间分布，我们使用了美国大气与海洋局（National Oceanic and Atmospheric Administration，缩写“NOAA”）发布的 500 米精度的夜晚灯光亮度数据与哥伦比亚大学发布的 2015 年度 1000 米精度的人口数据。由于夜晚灯光数据卫星过境时间是在凌晨 1:30，所以排除了暂时性光源的影响，能够较好地反应城市建设与工业发展水平。依据灯光强度，我们将全国按照 1km 栅格的精度划分为城乡两个等级，同时结合人口数据，可以算出某个行政区划内城市化人口的数量，据此我们可以计算出每个城市、区县的城市化人口比例。这种计算方式的优点是能够真正考察人口的城市化水平而不是城建区与总面积的比率，比如北京的行政区划内存在大量的山地、郊区等城市化水平低且人口密度低的区域，如果计算面积，北京的城市化水平并不高，但这显然不合理；本方法计算出的北京城市化率为 91.4%，即 91.4%

的北京人口居住在城市化的区域之中。

图 13 中展示了地级市层面城市化水平与数字金融覆盖广度和使用深度的关系。每一个散点代表一个城市，横轴是城市化水平，纵轴是数字金融覆盖广度指数（左图）和数字金融使用深度指数（右图）。从图 13 当中不难看出，无论覆盖广度还是使用深度，都与城市化水平高度正相关，城市化水平越高的地区，数字金融的覆盖广度和使用深度就越高。

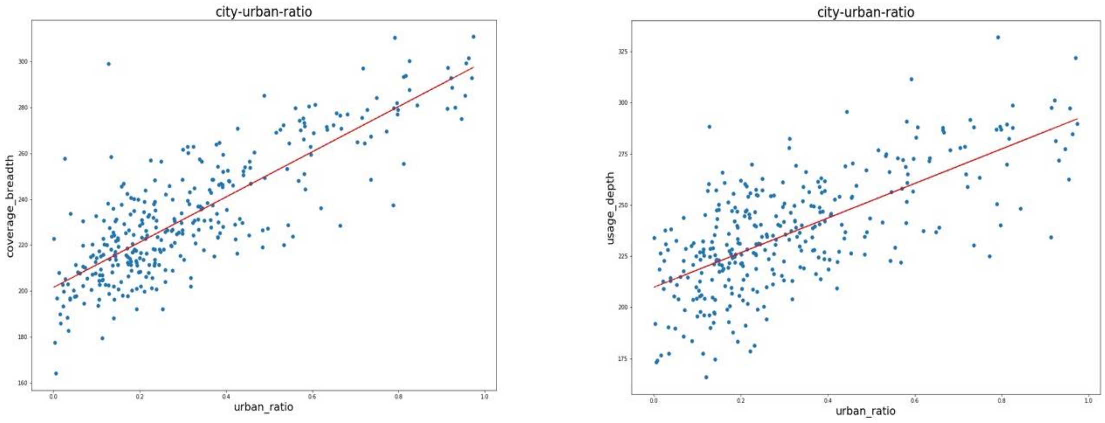

图 13：城市化水平与数字金融覆盖广度与使用深度

数据来源：北京大学数字普惠金融指数与哥伦比亚大学发布的人口栅格数据

分析城市化水平与数字金融发展之间的关系存在较强的内生性问题，比如，城市化水平与经济发展水平存在较强的相关性，即越发达的地区往往有更高的城市化水平，而发达地区的数字金融发展水平也越高，所以我们引入了另外一种直接度量某个空间内部人口聚集程度的指标：人口基尼系数，即以该地区每个 1km 栅格人口数量的基尼系数，取值为 0-1，越接近 1 则说明该区域内人口分布越集中；越接近 0 则该区域内的人口越分散。这一指标是人口聚集程度的直接度量，即一个地区人口基尼系数越大，人口分布越不平衡。图 14 展示了一个城市内部的人口基尼系数、城市化率与取对数之后的灯光亮度之间的散点图与线性拟合关系。我们使用灯光亮度来衡量一个地区的经济发展水平，可以发现，相较于城市化率与经济水平的关系（左图），人口基尼系数与城市经济发展水平（右图）的相关性更低。

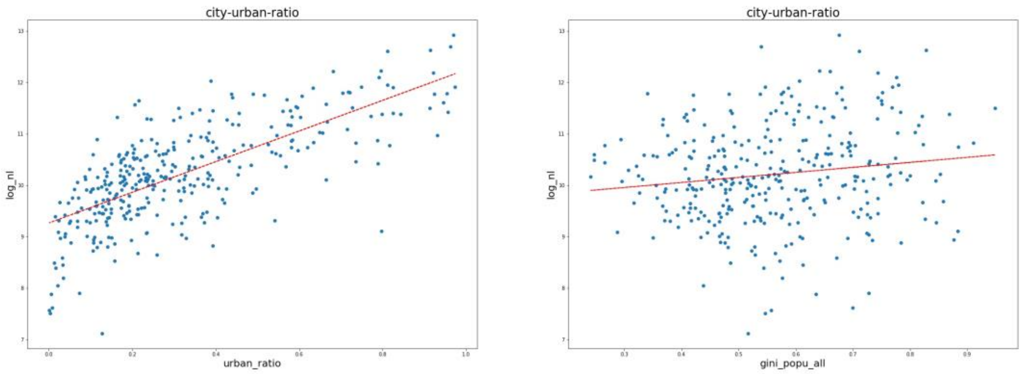

图 14：城市内部人口基尼系数与城市经济发展水平

数据来源：人口栅格数据与灯光栅格数据

我们在图 15 中展示了城市内部人口基尼系数与数字金融覆盖广度和使用深度的相关关系，可以发现，随着人口集中程度的增加，数字金融覆盖广度与使用深度均有所上升。

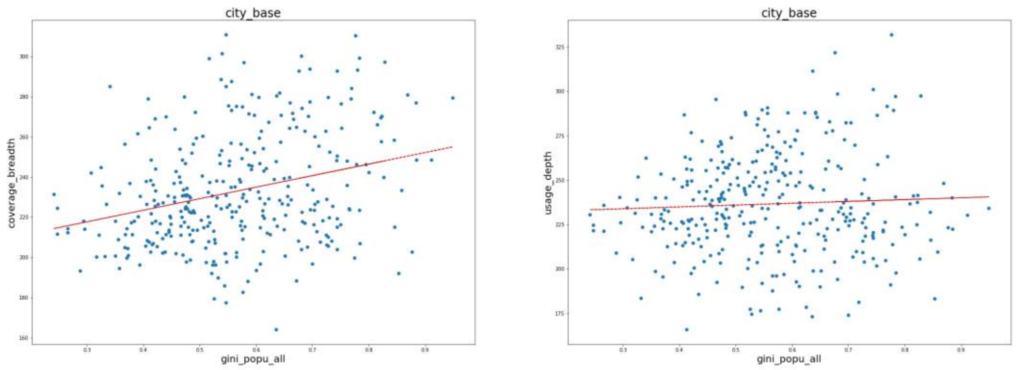

图 15：城市内部人口基尼系数与数字金融覆盖广度与使用深度

数据来源：人口栅格数据与北京大学数字普惠金融指数

为了分析城市内部人口聚集与数字普惠金融指数在不同地区的异质性关系，接下来我们将中国划分为三个区域，胡焕庸线以西（下简称西部）、胡焕庸线以东且秦岭淮河线以北（下简称北方）与胡焕庸线以东且秦岭淮河以南（下简称南方）进行分析。这样的划分比传统的南北方划分要更为合理，比如拉萨的纬度低于杭州，若单纯按照秦岭淮河线划分属于南方，但这显然是不合理的。

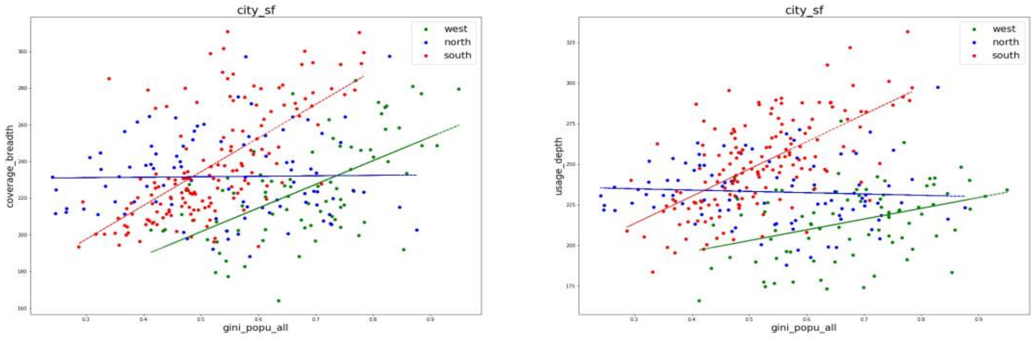

图 16：分地区人口基尼系数与数字金融覆盖广度与使用深度

数据来源：人口栅格数据与北京大学数字普惠金融指数

图 16 当中展现了西部（绿色点）、北方（蓝色点）与南方（红色点）的城市内部人口基尼系数与数字金融发展水平之间的关系，即不同区域人口集中程度与数字金融发展水平之间的关系。在这当中，最需要关注的一点是，在南方与西部，人口集中程度与数字金融发展水平（覆盖广度与使用深度）是正相关的，且南方这种正相关的趋势更强；但在北方，更集中的人口分布并未带来更高的数字金融发展水平。这一点值得我们关注，特别是考虑到如上文分析所发现的，2015 年后北方数字金融发展增速低于西部与南方。

为了更进一步分析城乡差异，我们使用区县层面的数据，并且，我们将城市化人口大于 70%且小于 95%的区县定义为城市，低于 30%的区县定义为县乡。城市化人口大于 95%的定义为核心区，核心区往往是那些面积较小、完全处于城区的区划，是一个城市的“纯蛋黄”位置。

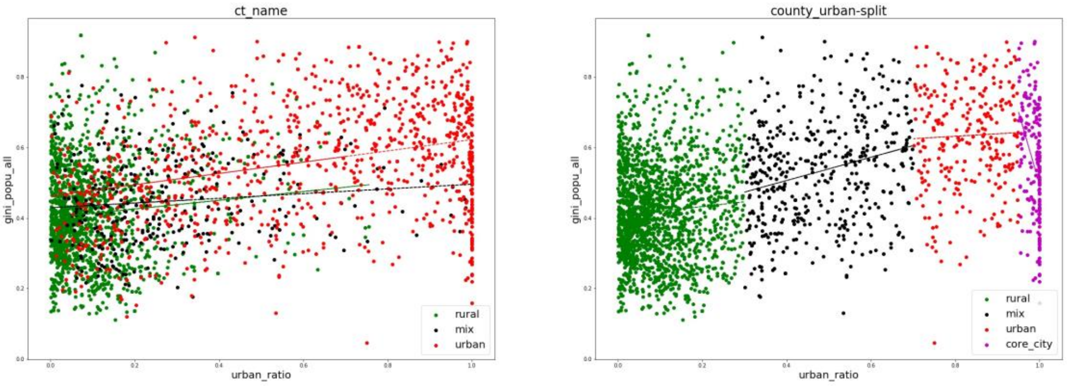

图 17：分地区人口基尼系数与城市化水平

如此定义的科学性如图 17 所示，图中每一个点为一个区县。左图展示了城市化率与人口基尼系数的相关关系，同时按照 2020 年行政区划代码命名规则划

分了城区（红色）县（绿色）与县级市（黑色），可以发现，由于近年大量县改区的存在，命名规则带来城乡划分的混乱。按照我们的划分方法的核心区（紫色）城（红色）乡（绿色）混合区（黑色）如右图所示。可以发现在核心区，城市化率的提高带来的是人口基尼系数的上升，而在其他地区，这种趋势相反。

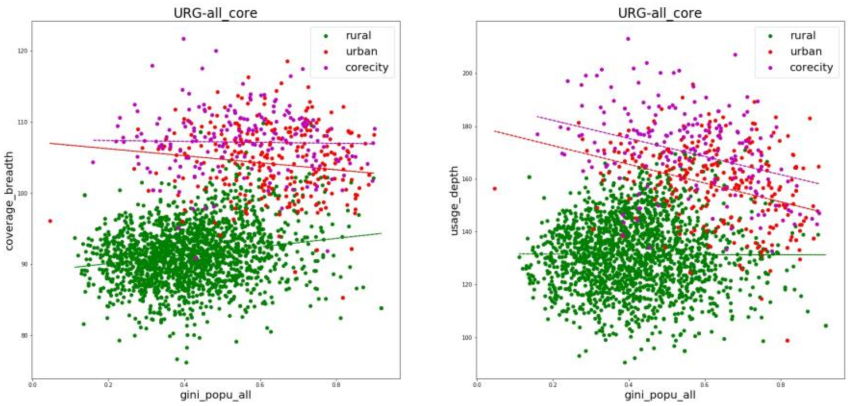

图 18：不同类型地区人口基尼系数与数字金融覆盖广度与使用深度

图 18 当中反映了城乡人口集中度与数字金融发展水平之间的关系，从中可以发现，在城市化水平较低的区县（图中绿点），人口集中度与数字经济发展水平正相关，而在城市与核心区，则为负相关。换言之，虽然从图 13 和图 15 当中来看，总体而言城市化水平（城市集中度）与数字金融发展正相关，但具体到发展水平已经很高的比较核心的都市化地区内部，其人口集中度与数字金融发展是负相关关系。

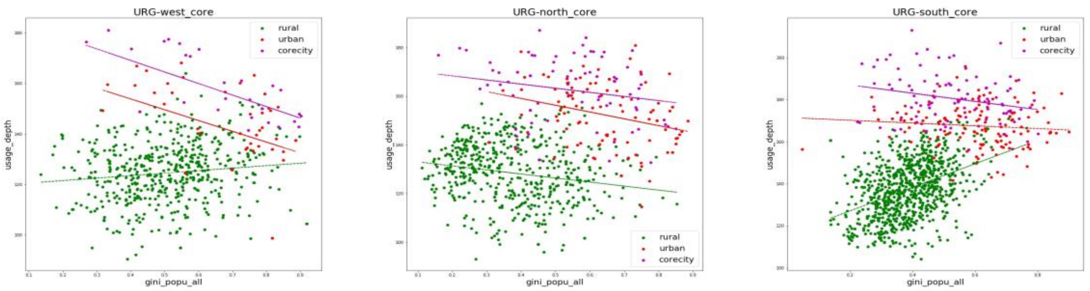

图 19：不同区域城市基尼系数与数字金融覆盖广度与使用深度

图 19 当中进一步分析了不同区域的城乡与数字金融发展程度之间的关系，左图为西部，中间图为北方，右图为南方。与地级市中的发现类似，只有在北方，人口集中没有带来更高水平的数字金融发展水平，不论是在城乡。在城市化和数字金融关系上，北方地区表现出了与其他地区非常不一样的特征。

四、结论

在本报告中，我们更新了“北京大学数字普惠金融指数”（2011-2020），分析了中国数字普惠金融的最新发展趋势，并重点讨论了数字普惠金融发展差距问题。得到如下几个主要结论：第一，经历了早期的高速发展后，数字普惠金融发展速度近几年有所放缓，但仍然维持了一个可观的增速，特别是新冠肺炎疫情之后，数字普惠金融发展依然增长，近十年来数字普惠金融的实践也初步证明了这种模式的可行性、可复制性，也显示出数字金融服务在疫情冲击下较强的韧性。第二，与前几年指数相比，最近几年指数增长的驱动力发生了非常明显的变化，数字金融使用深度的增长已经成为数字普惠金融指数增长的重要驱动力，这与第一期 2011-2015 年的指数形成鲜明对照，证明中国的数字普惠金融事业已经走过了粗放式的圈地时代，进入了有待深度拓展的新阶段。第三，前几年数字普惠金融区域间差异收敛速度明显，但近两年有所放缓，未来差异能否进一步缩小，主要体现在使用深度上的地区差异能否进一步缩小。第四，中国北方和中国南方的数字普惠金融发展水平差距由前两年的快速收窄，转为近两年的停滞不前，目前北方地区和南方地区的数字普惠金融发展差异，主要源于使用深度差距。第五，在城乡层面，总体而言，城市化水平与数字金融发展正相关，但在更细的视角上，在发展水平已经比较高的核心都市区内部，人口集中度与数字金融发展是负相关关系，即，数字金融发展水平在核心都市区的人口高度集中的区域反而较低。

数字金融是近年来中国社会各界广泛关注的领域，但基础数据的不完善极大地限制了相关领域的实证研究。这套指数为各界提供了一套反映数字普惠金融发展现状和演变趋势的基础数据。在本报告中，我们通过一些尽可能简化的方法，已经揭示出这套指数背后所能展现的丰富结论，我们相信在与家户、企业、地区特征等其他经济社会指标匹配之后，从这套指数当中还可以得到更多、更严谨的结论。当然，虽然我们对数字普惠金融的指标体系和指数编制方法进行了反复推敲，但其中存在的固有缺陷仍然必须承认。例如，限于数据的可获得性和不同机构数据的可匹配性等障碍，依然只能仅以一家代表性的数字金融机构为数据来源，因此不能反映数字普惠金融发展的完整图景。但如果将研究的问题限定于发展趋势、地区差距等，本套指数尚具有一定程度的代表性。特别是在缺乏度量地区间

数字普惠金融发展现状的情况下，本指数的发布可以为各界人士提供一个各地数字普惠金融发展程度的粗略度量，供关注该领域的各界人士参考。

参考文献

[1] 陈银娥、孙琼、徐文赟，《中国普惠金融发展的分布动态与空间趋同研究》，《金融经济学研究》，2015 年第 6 期，第 72-81 页。

[2] 郭峰，《数字经济在抗击新冠肺炎疫情中的作用与问题：一个文献综述》，《产业经济评论》，2021 年第 1 期，第 34-49 页。

[3] 郭峰、孔涛、王靖一、张勋、程志云、阮方圆、孙涛、王芳，《北京大学数字普惠金融指数（2011-2015）》，北京大学数字金融研究中心，2016 年 7 月。

[4] 郭峰、王靖一、程志云、李勇国、王芳、魏爱勇，《北京大学数字普惠金融指数（2011-2018）》，北京大学数字金融研究中心，2019 年 5 月。

[5] 郭峰、王靖一、王芳、孔涛、张勋、程志云，《测度中国数字普惠金融发展: 指数编制与空间特征》，《经济学季刊》，2020 年第 19 卷第 4 期，第 1401-1418 页。

[6] 黄益平、黄卓，“中国的数字金融发展：现在与未来”，《经济学季刊》, 2018 年第 1 卷第 4 期，第 205-218 页。

[7] 焦瑾璞、黄亭亭、汪天都、张韶华、王瑱，《中国普惠金融发展进程及实证研究》，《上海金融》，2015 年第 4 期，第 12-22 页。

[8] 彭非、袁卫、惠争勤，《对综合评价方法中指数功效函数的一种改进探讨》,《统计研究》，2007 年第 12 期，第 29-34 页。

[9] 世界银行集团，《2014 年全球金融发展报告：普惠金融》，北京：中国财政经济出版社，2015 年 10 月。

[10] 王伟、田杰、李鹏，《我国金融排除度的空间差异及影响因素分析》，《西南金融》，2011 年第 3 期，第 14-17 页。

[11] 王靖一、郭峰、李振华、王芳、蒋正伟、李勇国，《数字经济助力中国东西部经济平衡发展》，北京大学数字金融研究中心研究报告，2019 年 9 月。

[12] 伍旭川、肖翔，《基于全球视角的普惠金融指数研究》，《南方金融》，2014 年第 6 期，

[13] 中国人民银行金融消费权益保护局，《2017 年中国普惠金融指标分析报告》，中国人民银行研究报告，2018 年 8 月。

[14] Barro, R. J., and X. Sala-i-Martin, “Convergence”, Journal of Political Economy, 1992, 100(2), 223-251.

[15] Sala-I-Martin X., “The Classical Approach to Convergence Analysis”, Economic Journal 1996, 106(437), 1019-1036.

[16] Sarma, M., 2012, “Index of Financial Inclusion-A measure of financial sector inclusiveness”, Berlin Working Papers on Money, Finance, Trade and Development No. 07/2012.

[17] Xiao, K., 2020, “Saving Lives Versus Saving Livelihoods: Can Big Data Technology Solve the Pandemic Dilemma?”, Working Paper, Available at: https://ssrn.com/abstract=3583919.

附录 1：省级数字普惠金融指数（2011-2020）

附表 1：2011 年数字普惠金融指数及分类指数

行政区划	总指数	覆盖广度	使用深度	支付	保险	信贷	数字化程度
北京市	79.41	97.53	72.23	79.4	59.48	76.75	32.59
天津市	60.58	69.37	53.33	55.52	75.74	43.73	44.72
河北省	32.42	18.46	44.19	24.36	24.83	54.49	57.15
山西省	33.41	28.94	21.61	19.48	20.25	22.42	69.57
内蒙古	28.89	24.65	30.27	27.51	49.83	22.42	40.35
辽宁省	43.29	44.96	44.64	49.24	48.98	42.31	35.33
吉林省	24.51	23.75	24.04	23.89	23.85	24.13	27.86
黑龙江省	33.58	21.12	36.28	33.74	39.37	35.28	69.83
上海市	80.19	98.85	86.24	100	72.82	90.3	7.58
江苏省	62.08	66.7	79.22	80.77	78.49	79.35	15.71
浙江省	77.39	85.53	93.52	96.52	100	90.48	21.22
安徽省	33.07	20.2	55.58	49.04	40.63	62.54	34.66
福建省	61.76	63.28	68.51	77.26	46.12	76.87	44.5
江西省	29.74	13.97	54.82	56.7	46.6	58.04	36.21
山东省	38.55	33.67	47.16	44.24	9.07	63.38	39.01
河南省	28.4	13.54	38.11	38.37	0.25	53.88	59.81
湖北省	39.82	35.17	53.56	69.15	44.82	55.47	30.18
湖南省	32.68	15.33	60.73	53.36	51.76	65.29	39.02
广东省	69.48	63.41	80.97	59.96	50.69	95.96	68.66
广西	33.89	19.98	44.06	52.17	42.03	44	61.33
海南省	45.56	30.96	57.74	60.56	61.02	56.06	71.63
重庆市	41.89	40.38	47.46	59.24	57.04	42.14	36.77
四川省	40.16	29.02	58.56	45.49	73.61	53.74	43.5
贵州省	18.47	3.06	27.51	49.21	47.38	16.79	52.92
云南省	24.91	7.47	48.39	55.11	82.08	33.58	39.81
西藏	16.22	3.37	30.16	0	66.3	18.46	33.33
陕西省	40.96	37.81	29.74	34.44	26.16	30.71	71.74
甘肃省	18.84	4.99	12.76	10.82	6.48	15.59	75.61
青海省	18.33	1.96	6.76	0	21.99	1.16	93.42
宁夏	31.31	32.27	23.16	15.68	41.09	16.51	42.96
新疆	20.34	12.92	23.6	21.47	51.84	12.05	38.92

附表 2：2012 年数字普惠金融指数及分类指数

行政区划	总指数	覆盖广度	使用深度	支付	保险	信贷	数字化程度
北京市	150.65	155.56	159.42	110.02	216.68	141.07	118.47
天津市	122.96	110.61	135.77	74.34	212.58	110.61	140.44
河北省	89.32	65.46	108.15	43.52	132.99	105.02	133.9
山西省	92.98	75.2	86.48	37.43	143.54	68.17	163.5
内蒙古	91.68	75.03	95.44	45.63	158.4	74.75	139.78
辽宁省	103.53	89.01	120.36	69.54	187.08	98.21	120.91
吉林省	87.23	69.43	93.83	44.98	127.45	85.27	133.99
黑龙江省	87.91	66.48	100.46	56.37	153.19	83.4	135.89
上海市	150.77	149.35	174.72	136.14	227.7	156.94	111.94
江苏省	122.03	106.69	156.55	98.32	228.07	133.23	109.94
浙江省	146.35	128.5	200.42	130.89	324.29	156.52	107.07
安徽省	96.63	66.06	138.06	67.84	215.71	113.52	122.31
福建省	123.21	112.74	140.25	100.51	169.52	132.49	126.79
江西省	91.93	59.82	132.68	75.05	207.3	108	123.92
山东省	100.35	80.15	127.53	61.65	176.43	114.51	117.68
河南省	83.68	61.93	98.07	61.8	104.06	99.63	129.37
湖北省	101.42	82.06	125.84	90.83	173.75	109.77	121
湖南省	93.71	63.39	132.38	75.16	204.99	108.49	123.56
广东省	127.06	111.37	149.38	81.73	153.26	155.34	138.31
广西	89.35	66.47	104.58	69.07	142.54	92.72	137.25
海南省	102.94	79.51	120.72	87.09	169.69	104.05	147.98
重庆市	100.02	85.39	116.14	89.6	180.84	92.12	119.05
四川省	100.13	74.36	126.5	78.66	179.64	109.68	137.31
贵州省	75.87	49.87	89.92	79.69	155.99	63.49	136.21
云南省	84.43	52.78	111.96	75.43	177.58	88.67	138.91
西藏	68.53	32.86	71.07	16.85	164.95	37.98	181.65
陕西省	98.24	83.62	98.61	58.38	141.46	85.23	145.88
甘肃省	76.29	54.72	68.98	36.32	117.06	52.57	160.79
青海省	61.47	47.12	51.85	16.33	136.35	20.57	126.3
宁夏	87.13	76.78	90.34	40.51	158.36	67.53	115.46
新疆	82.45	60.88	85.14	46.28	159.44	58.5	148.76

附表 3：2013 年数字普惠金融指数及分类指数

行政区划	总指数	覆盖广度	使用深度	支付	保险	货基	信贷	数字化程度
北京市	215.62	193.86	247.5	136.53	617.63	94.21	131.17	229.57
天津市	175.26	146.54	197.52	110.17	511.81	53.85	100.24	229.67
河北省	144.98	105.66	162.85	64.92	422.49	24.22	88.71	242.35
山西省	144.22	115.4	139.08	61.87	422.74	14.73	50.21	248.75
内蒙古	146.59	116.37	138.84	73.66	369.88	4.22	72.3	260.45
辽宁省	160.07	126.67	181.54	93.89	485.17	27.93	90.42	231.33
吉林省	138.36	106.85	147.95	72.92	396.34	8.92	76.01	224.97
黑龙江省	141.4	104.49	152.58	80.25	418.82	13.87	72.84	242.97
上海市	222.14	187.31	280.93	169.95	680.74	100	156.87	230.3
江苏省	180.98	144.68	223.09	128.77	543.88	70.02	125.46	224.3
浙江省	205.77	167.96	265.48	166.23	649.99	95.21	144.69	222.12
安徽省	150.83	106.51	190.86	95.33	493.54	37.52	100.96	224.45
福建省	183.1	157.43	194.12	130.14	436.21	60.94	122.6	247.85
江西省	146.13	99.81	183.73	102.46	478.45	43.19	93.4	230.78
山东省	159.3	122.01	189.07	89.87	483.75	45.87	101.22	228.32
河南省	142.08	105.06	155.23	87.45	395.02	31.51	83.51	240.42
湖北省	164.76	123.74	197.04	111.2	511.31	53.97	99.5	241.51
湖南省	147.71	103.46	175	100.22	454.11	34.87	90.4	244.25
广东省	184.78	153.33	208.44	106.47	466.62	59.28	137.12	245.61
广西	141.46	106.97	153.84	99.06	417	20.3	72.55	232.82
海南省	158.26	121.75	173.37	110.76	468.48	32.71	80.81	251.39
重庆市	159.86	125.27	178.2	113.4	471.66	35.23	86.97	240.74
四川省	153.04	114.03	176.71	102.38	445.78	35.62	96.41	238.82
贵州省	121.22	89.59	125.46	100.53	384.99	0	41	217.93
云南省	137.9	95.59	153.55	97.13	413.08	26.56	72.86	249.15
西藏	115.1	74.09	112.84	63.75	363.27	21.4	29.17	254.65
陕西省	148.37	123.6	145.94	78.61	399.93	23.61	68	234.55
甘肃省	128.39	96.77	114.2	69.51	357.1	3.33	36.43	258.6
青海省	118.01	88.18	113.42	63.67	389.3	3.26	22.35	224.82
宁夏	136.74	115.08	129.02	44.5	399.35	3.37	46.74	222.32
新疆	143.4	101.44	146.39	76.03	422.5	24.01	59.57	276.48

附表 4：2014 年数字普惠金融指数及分类指数

行政区划	总指数	覆盖广度	使用深度	支付	保险	货基	投资	信贷	数字化程度
北京市	235.36	243.92	219.89	196.25	663.95	209.83	94.93	120.76	235.22
天津市	200.16	193.86	180.28	165.52	576.75	166.06	59.55	97.95	257.11
河北省	160.76	149.97	131.34	114.97	423.99	126.08	27.5	79.94	249.86
山西省	167.66	163.16	124.78	117.06	462.67	122.03	20.44	53.43	260.43
内蒙古	172.56	165.46	114.88	117.47	428.7	111.48	9.2	53.41	300.84
辽宁省	187.61	175.49	162.89	143.45	550.31	139.88	40.66	87.32	272.53
吉林省	165.62	154.91	136.01	118.63	462.03	119.15	30.66	73.74	254.75
黑龙江省	167.8	152.48	142.48	127.57	484.01	129.53	32.27	75.98	264.41
上海市	239.53	237.02	242.78	225.1	725.26	220.53	96.62	142.88	241.88
江苏省	204.16	193.18	201.09	184.42	611.07	182.13	72.09	119.53	246.02
浙江省	224.45	217.48	233.67	224.06	687.54	206.83	96.58	139.65	230.71
安徽省	180.59	156.56	173.84	151.5	547.03	151.28	59.96	98.99	272.22
福建省	202.59	204.22	164.85	181.25	457.02	161.83	37.59	124.93	265.76
江西省	175.69	148.73	167.19	154.14	535.69	148.08	44.25	98.59	280.18
山东省	181.88	169.89	161.19	142.07	511.64	151.85	49.31	91.92	259.08
河南省	166.65	157.52	132.24	137.08	415.49	136.22	23.47	84.05	259.31
湖北省	190.14	176.61	175.7	165.88	548.79	162.44	59.01	99.75	261.07
湖南省	167.27	150.42	153.46	148.44	479.47	139.81	40.84	94.02	247.99
广东省	201.53	199.63	175.04	156.69	485.07	159.13	56.45	128.04	255.98
广西	166.12	154.29	139.98	139.02	449.06	115.7	36.92	82.67	252.66
海南省	179.62	170.99	153.8	157.71	529.76	136.61	42.99	71.93	255.03
重庆市	184.71	175.57	157.88	158.33	514.01	141.85	34.8	92.5	263.63
四川省	173.82	162.58	159.82	149.32	499.44	140.04	46.33	96.92	236.39
贵州省	154.62	139.9	114.08	126.53	421.51	95.77	10.18	55.52	276.9
云南省	164.05	147.22	144.3	134.42	475.79	123.72	35.1	82.03	255.54
西藏	143.91	126.67	108.76	113.51	450.07	126.67	19.73	21.11	264.7
陕西省	178.73	173.25	139	122.05	472.64	131.49	32.45	72.71	269
甘肃省	159.76	148.1	107.29	111.91	426.42	110.28	11.49	35.84	293.6
青海省	145.93	139.24	108.4	109.16	443.05	111.57	22.21	24.58	236.23
宁夏	165.26	167.18	114.28	111.45	446.04	111.85	14.42	41.97	251.55
新疆	163.67	151.28	134.87	133.48	487.16	134.97	39.1	50.72	256.91

附表 5：2015 年数字普惠金融指数及分类指数

行政区划	总指数	覆盖广度	使用深度	支付	保险	货基	投资	信贷	信用	数字化程度
北京市	276.38	268.39	234.17	243.23	469.42	239.07	230.65	173.81	92.32	379.48
天津市	237.53	211.89	195.46	206.76	427.33	193.62	183.24	143.47	52.11	398.62
河北省	199.53	172.78	151.45	161.59	306.8	162.76	144.95	127.69	0	375.2
山西省	206.3	186.14	141.52	159.41	337.67	156.65	124.49	97.75	21.99	390.57
内蒙古	214.55	185.34	136.04	154.71	332.6	136.78	111.59	100.99	10.04	453.66
辽宁省	226.4	194.17	178.41	181.6	398.6	168.45	166.5	132.75	37.34	420.06
吉林省	208.2	175.49	154.68	166.01	343.1	152.29	149.57	113.84	20.53	413.47
黑龙江省	209.93	174.68	164.06	170.1	363.16	163.96	168.8	111.5	33.75	409.72
上海市	278.11	258.98	259.81	268.49	521.32	252.66	246.52	201.7	99.92	374.54
江苏省	244.01	215.94	218.62	227.43	441.96	216.8	196.83	173.28	88.44	382.84
浙江省	264.85	239.33	251.29	270.92	518.33	243.83	233.82	196.9	74.43	373.77
安徽省	211.28	171.65	189.78	196.92	396.7	188.13	175.28	149.57	48.54	381.23
福建省	245.21	226.6	198.23	230.19	396.13	198.59	140.07	185.79	62.59	392.01
江西省	208.35	170.86	182.48	194.78	397.91	182.27	144.49	151.45	48.25	379.14
山东省	220.66	192.11	178.15	186.17	372.83	187.49	165.04	141.22	33.02	392.16
河南省	205.34	181.5	151.05	174.98	307.69	171.16	127.15	130.9	15.71	382.73
湖北省	226.75	199.53	189.08	210.98	396.45	196.58	168.41	146.92	57.81	385.07
湖南省	206.38	170.07	174.47	186.49	371.97	171.84	150.14	143.89	34.65	384.24
广东省	240.95	225.52	195.87	207.36	365.29	194.22	155.49	187.62	55.21	373.79
广西	207.23	176.33	153.46	177.23	341.68	145.27	108.67	133.25	38.33	406.94
海南省	230.33	192.26	184.91	195.59	438.8	164.35	163.58	128.84	57.04	438.59
重庆市	221.84	197.46	171.58	191.7	395.35	168.62	137.05	132.4	44.92	393.65
四川省	215.48	182.08	176.54	190.6	378.83	168.17	152.43	141.43	48.59	396.51
贵州省	193.29	160.98	132.74	155.83	345.19	119.99	99.82	95.4	17.94	410.01
云南省	203.76	167.96	158.79	168.47	383.48	144.2	126.27	122.14	27.86	403.67
西藏	186.38	139.87	157.75	168.77	447.65	160.9	131.77	87.66	22.33	391.97
陕西省	216.12	194.92	157.95	162.76	355.33	161.98	142.39	116.87	35.15	391.85
甘肃省	199.78	169.67	125.25	142.62	319.06	130.5	107.17	84.74	6.12	434.64
青海省	195.15	159.59	136.5	143.26	357.89	135.74	125.92	85.05	4.95	419.14
宁夏	214.7	190.35	134.87	149.39	364.97	135.96	104.12	90.12	9.68	440.18
新疆	205.49	172.01	148.6	165.46	397.95	155.11	125.97	92.83	10.14	419.4

附表 6：2016 年数字普惠金融指数及分类指数

行政区划	总指数	覆盖广度	使用深度	支付	保险	货基	投资	信贷	信用	数字化程度
北京市	286.37	285.65	263.74	286.87	566.47	262.63	210.94	172.79	251.61	329.90
天津市	245.84	225.41	231.61	247.13	541.83	226.72	159.76	155.95	202.60	339.15
河北省	214.36	191.55	196.87	214.64	434.79	213.26	125.48	145.45	174.72	321.46
山西省	224.81	205.51	189.38	206.37	451.07	201.16	101.35	134.39	187.73	352.96
内蒙古	229.93	202.00	184.89	195.22	466.55	176.23	99.44	125.88	176.30	404.00
辽宁省	231.41	207.74	220.06	219.02	523.97	205.06	142.03	152.90	197.61	330.21
吉林省	217.07	191.94	204.14	209.46	482.14	195.40	124.46	144.93	189.90	323.59
黑龙江省	221.89	191.24	206.54	214.35	496.97	204.83	122.77	142.58	195.35	350.97
上海市	282.22	274.25	281.48	309.09	615.25	278.98	222.35	190.79	233.86	309.94
江苏省	253.75	233.22	253.08	279.69	560.71	255.34	172.07	174.22	253.81	322.80
浙江省	268.10	254.44	270.62	316.53	608.21	279.39	192.47	189.77	212.02	308.66
安徽省	228.78	194.89	229.95	248.77	524.77	234.42	148.09	161.80	214.37	338.54
福建省	252.67	240.47	245.12	287.16	566.68	249.60	137.36	180.46	228.43	306.70
江西省	223.76	188.79	222.74	242.01	533.27	226.68	129.04	157.10	202.21	341.08
山东省	232.57	209.80	217.81	235.54	488.04	228.83	145.30	154.33	196.47	334.58
河南省	223.12	200.65	199.22	226.02	446.66	219.76	113.26	146.68	195.95	340.80
湖北省	239.86	215.55	233.41	264.87	530.70	241.57	158.89	159.41	210.13	331.83
湖南省	217.69	186.13	219.80	233.88	517.19	216.16	130.90	158.56	198.54	318.07
广东省	248.00	240.07	236.50	266.79	516.88	238.18	143.89	181.38	217.88	295.07
广西	223.32	193.51	202.21	227.42	476.80	200.46	101.20	149.01	210.69	360.15
海南省	231.56	210.09	220.35	232.99	562.52	203.50	140.08	134.72	208.30	322.83
重庆市	233.89	214.03	211.54	236.07	508.19	209.84	120.02	147.53	202.76	340.10
四川省	225.41	197.00	216.54	237.79	492.85	213.79	135.37	154.36	209.48	335.38
贵州省	209.45	180.70	182.70	190.36	479.22	168.13	74.26	131.03	184.71	353.03
云南省	217.34	185.37	203.17	206.68	501.83	186.39	116.03	141.41	190.33	348.65
西藏	204.73	167.21	202.53	210.12	537.69	197.95	141.09	113.15	163.39	332.66
陕西省	229.37	211.17	202.87	213.12	471.15	203.41	128.32	140.55	195.17	337.60
甘肃省	204.11	189.28	172.66	182.10	434.02	172.07	99.70	111.11	170.03	310.24
青海省	200.38	177.73	182.26	181.72	457.67	173.70	112.73	118.71	165.84	308.11
宁夏	212.36	205.92	179.62	191.98	461.68	174.42	91.80	118.95	179.54	293.12
新疆	208.72	190.32	190.11	197.22	490.82	189.52	130.24	112.29	155.41	303.31

附表 7：2017 年数字普惠金融指数及分类指数

行政区划	总指数	覆盖广度	使用深度	支付	保险	货基	投资	信贷	信用	数字化程度
北京市	329.94	316.12	357.24	303.12	717.79	274.60	388.93	210.41	340.35	326.02
天津市	284.03	257.90	310.13	257.20	666.47	245.04	312.83	188.37	265.14	322.91
河北省	258.17	232.89	273.45	226.55	572.54	222.53	263.34	170.23	269.07	313.87
山西省	259.95	243.02	254.98	216.39	553.60	220.15	234.64	159.25	234.50	324.92
内蒙古	258.50	238.92	249.20	196.40	563.67	190.60	240.18	141.03	243.89	340.10
辽宁省	267.18	239.87	291.27	224.18	630.04	218.81	282.88	182.83	261.75	313.57
吉林省	254.76	227.45	273.62	210.33	593.75	209.98	262.23	170.99	251.91	310.72
黑龙江省	256.78	226.00	275.86	214.68	593.53	217.39	261.07	173.30	262.03	323.77
上海市	336.65	305.89	396.05	333.43	785.39	294.74	398.99	231.81	486.77	330.31
江苏省	297.69	272.32	328.93	298.29	688.84	276.23	318.37	205.11	301.74	324.69
浙江省	318.05	290.06	366.40	343.86	768.73	303.17	344.68	224.29	372.47	322.66
安徽省	271.60	234.70	309.55	271.32	662.72	260.40	282.60	188.94	322.57	324.48
福建省	299.28	275.40	334.33	309.03	704.80	292.68	300.31	210.30	340.23	314.47
江西省	267.17	228.52	305.92	261.96	672.55	257.50	272.23	182.36	327.53	324.38
山东省	272.06	247.19	290.92	248.98	619.98	244.43	282.61	180.27	257.85	319.92
河南省	266.92	241.45	279.56	247.49	584.25	242.81	258.81	170.34	300.21	328.09
湖北省	285.28	253.63	317.58	290.35	652.84	269.86	311.25	192.90	317.58	331.10
湖南省	261.12	223.47	297.70	248.52	641.34	246.38	280.06	181.95	290.21	318.96
广东省	296.17	275.91	328.17	288.57	676.58	267.20	297.05	209.91	358.26	304.92
广西	261.94	232.73	279.52	244.56	601.91	230.97	247.06	174.67	293.37	326.44
海南省	275.64	253.39	297.53	247.67	656.86	237.26	292.47	171.45	279.20	309.34
重庆市	276.31	249.50	301.21	246.62	621.74	233.38	285.95	179.45	360.08	319.57
四川省	267.80	231.87	301.54	245.24	620.25	237.72	298.90	181.94	321.86	325.14
贵州省	251.46	227.77	258.44	207.60	594.73	201.57	226.99	157.08	246.54	316.99
云南省	256.27	223.54	282.85	217.82	604.28	211.19	296.57	164.67	261.55	316.08
西藏	245.57	209.29	273.79	219.80	596.15	226.33	297.58	146.69	239.68	314.10
陕西省	266.85	246.48	276.00	226.87	586.20	221.87	276.76	166.68	253.10	317.47
甘肃省	243.78	227.38	240.39	196.94	526.80	191.33	243.43	132.77	237.38	304.10
青海省	240.20	215.67	251.09	189.51	534.91	195.47	278.41	135.86	232.68	301.42
宁夏	255.59	242.42	252.21	189.87	552.15	185.65	239.44	138.21	310.56	305.24
新疆	248.69	228.82	249.10	196.68	539.02	203.00	279.76	131.99	209.86	313.56

附表 8：2018 年数字普惠金融指数及分类指数

行政区划	总指数	覆盖广度	使用深度	支付	保险	货基	投资	信贷	信用	数字化程度
北京市	368.54	353.87	366.78	317.54	747.90	229.24	405.88	216.26	345.81	420.19
天津市	316.88	295.35	317.94	278.23	717.91	201.49	294.27	191.02	316.05	386.10
河北省	282.77	264.06	267.92	238.96	608.74	178.16	229.24	166.12	280.18	371.55
山西省	283.65	277.03	249.73	243.58	560.07	183.78	192.97	163.01	273.11	367.19
内蒙古	271.57	269.49	232.31	205.87	561.44	138.36	178.88	141.03	261.41	349.76
辽宁省	290.95	271.81	279.48	237.75	647.87	165.28	241.64	169.26	298.84	375.01
吉林省	276.08	256.55	255.23	216.84	584.77	154.24	214.50	159.50	278.48	378.46
黑龙江省	274.73	256.12	254.88	220.17	571.27	167.34	213.10	160.68	285.72	372.28
上海市	377.73	346.33	400.40	356.14	849.62	261.16	419.90	243.08	344.98	440.26
江苏省	334.02	311.95	333.09	313.48	732.70	234.92	302.17	208.82	319.52	408.62
浙江省	357.45	330.17	372.01	379.51	838.08	256.93	337.91	229.70	328.80	421.07
安徽省	303.83	273.41	309.62	286.38	726.16	220.86	254.70	191.80	299.98	393.79
福建省	334.44	312.31	334.30	324.73	748.45	233.93	282.49	215.68	325.32	407.76
江西省	296.23	266.46	296.52	273.50	688.64	209.01	240.49	186.82	296.59	394.00
山东省	301.13	281.99	287.85	263.14	653.12	200.62	245.07	179.46	293.13	388.48
河南省	295.76	278.46	275.74	269.68	627.45	205.03	217.08	178.31	281.95	389.27
湖北省	319.48	292.56	322.44	307.65	705.24	226.21	298.14	199.53	310.68	402.99
湖南省	286.81	258.07	286.55	248.24	653.41	192.38	246.43	178.89	290.13	382.19
广东省	331.92	312.44	329.93	305.54	733.02	215.65	282.15	214.14	332.90	399.86
广西	289.25	270.41	272.49	258.82	629.05	176.50	206.85	177.77	297.43	381.93
海南省	309.72	294.40	300.23	265.85	683.33	183.76	268.65	184.31	300.74	377.54
重庆市	301.53	285.11	285.60	261.95	622.31	181.26	255.20	178.41	311.21	384.74
四川省	294.30	266.15	295.83	256.25	656.64	188.36	275.92	177.18	309.53	384.51
贵州省	276.91	267.39	241.33	220.88	575.72	144.55	163.71	161.93	276.39	373.01
云南省	285.79	262.29	278.84	228.89	622.58	161.74	266.65	166.04	288.61	376.06
西藏	274.33	249.82	267.16	234.36	582.08	190.52	262.27	152.56	278.34	368.33
陕西省	295.95	281.05	277.15	253.23	609.53	181.85	243.87	173.95	295.99	379.31
甘肃省	266.82	261.29	227.52	207.77	511.72	145.81	189.66	142.63	254.10	356.54
青海省	263.12	251.69	235.31	194.60	505.29	148.52	226.63	141.36	258.52	351.43
宁夏	272.92	274.25	225.27	207.53	522.09	133.39	168.32	144.76	268.43	355.14
新疆	271.84	267.35	232.94	209.70	491.33	157.37	231.08	135.86	254.82	357.37

附表 9：2019 年数字普惠金融指数及分类指数

行政区划	总指数	覆盖广度	使用深度	支付	保险	投资	信贷	数字化程度
北京市	399.00	384.66	402.07	330.51	803.14	470.55	253.17	440.83
天津市	344.11	323.86	349.01	292.57	775.65	342.68	224.82	402.11
河北省	305.06	284.39	297.33	258.60	654.05	278.90	200.68	387.38
山西省	308.73	305.61	277.89	265.71	611.36	238.93	192.51	375.07
内蒙古	293.89	291.45	260.31	224.23	636.92	211.50	166.98	362.98
辽宁省	311.01	292.44	302.52	249.44	700.18	275.39	194.63	387.77
吉林省	292.77	275.75	275.93	233.29	633.62	241.92	182.76	379.62
黑龙江省	292.87	275.79	274.64	237.00	619.68	244.87	181.23	382.41
上海市	410.28	378.25	439.91	365.22	932.26	480.11	282.23	462.23
江苏省	361.93	341.50	365.50	324.04	789.04	354.61	245.29	422.92
浙江省	387.49	362.40	404.65	363.10	899.85	392.67	265.62	439.16
安徽省	330.29	301.15	341.53	304.14	773.50	314.18	225.11	406.11
福建省	360.51	340.65	363.73	335.16	788.30	339.13	247.17	420.25
江西省	319.13	294.32	319.18	288.49	711.20	288.25	213.15	400.97
山东省	327.36	309.97	318.54	279.69	703.74	294.69	214.60	400.84
河南省	322.12	309.34	301.85	293.00	654.00	260.81	214.63	401.16
湖北省	344.40	320.79	348.53	318.56	733.52	352.25	230.64	414.89
湖南省	310.85	282.28	311.81	272.33	690.35	296.92	203.99	403.46
广东省	360.61	339.98	364.50	321.68	788.90	342.19	250.38	421.66
广西	309.91	295.26	292.48	275.08	667.03	240.81	198.27	390.01
海南省	328.75	319.41	314.46	282.29	701.15	294.02	201.64	385.58
重庆市	325.47	311.03	310.36	272.38	685.77	293.35	203.75	400.62
四川省	317.11	291.22	319.53	274.15	705.33	320.67	201.32	398.23
贵州省	293.51	292.66	245.66	239.93	597.01	159.88	172.66	383.30
云南省	303.46	284.43	291.12	243.39	647.40	281.57	185.35	388.74
西藏	293.79	271.14	293.21	249.17	643.68	311.70	170.02	369.65
陕西省	322.89	308.21	309.14	279.69	660.36	295.75	209.58	396.36
甘肃省	289.14	287.31	251.74	236.24	558.19	216.91	171.55	363.16
青海省	282.65	272.90	252.75	222.00	535.43	243.30	168.22	369.19
宁夏	292.31	299.04	241.55	227.62	545.37	196.39	166.49	362.35
新疆	294.34	293.48	256.31	232.35	557.77	251.99	160.13	366.30

附表 10：2020 年数字普惠金融指数及分类指数

行政区划	总指数	覆盖广度	使用深度	支付	保险	投资	信贷	数字化程度
北京市	417.88	397.00	445.83	342.69	787.94	646.97	265.33	436.02
天津市	361.46	340.29	373.91	291.87	710.85	472.15	242.45	408.74
河北省	322.70	304.10	318.42	268.50	625.02	357.46	222.89	391.92
山西省	325.73	327.29	291.37	272.44	571.95	287.49	220.11	383.04
内蒙古	309.39	310.40	275.66	244.52	583.57	263.63	198.35	367.40
辽宁省	326.29	307.11	328.12	259.31	677.78	364.96	218.37	386.33
吉林省	308.26	290.78	297.63	245.12	640.01	294.01	209.74	385.29
黑龙江省	306.08	290.48	293.69	241.55	615.42	301.91	202.65	380.09
上海市	431.93	395.20	488.68	368.97	945.37	658.18	296.03	450.08
江苏省	381.61	362.11	395.01	327.20	752.29	482.20	262.86	421.70
浙江省	406.88	382.07	439.25	365.55	882.00	531.88	281.25	429.98
安徽省	350.16	323.75	366.15	309.34	737.67	418.40	245.22	408.38
福建省	380.13	359.21	401.80	341.23	753.13	494.23	266.99	409.82
江西省	340.61	316.14	353.23	295.46	690.90	419.54	232.88	398.52
山东省	347.81	331.66	343.49	287.68	673.67	394.06	234.73	409.00
河南省	340.81	331.16	321.21	298.94	602.41	343.53	239.88	408.32
湖北省	358.64	336.54	369.58	308.96	672.10	478.21	241.33	411.73
湖南省	332.03	302.28	347.44	281.54	672.24	431.40	222.62	402.30
广东省	379.53	356.94	404.35	329.03	757.80	505.98	267.80	409.06
广西	325.17	311.98	313.24	279.42	618.87	344.61	215.71	390.41
海南省	344.05	335.87	337.24	287.31	659.71	405.14	215.24	383.46
重庆市	344.76	329.39	343.91	274.10	689.03	413.42	219.24	397.12
四川省	334.82	310.76	344.86	277.90	660.95	431.17	220.40	396.05
贵州省	307.94	313.24	258.20	246.72	571.73	215.78	188.99	380.81
云南省	318.48	302.46	309.45	255.61	605.03	363.33	205.04	387.78
西藏	310.53	290.18	319.38	262.93	598.33	425.11	190.74	361.67
陕西省	342.04	329.53	331.73	285.89	628.70	389.76	227.29	402.11
甘肃省	305.50	308.87	265.35	250.23	515.02	271.34	194.52	367.36
青海省	298.23	292.06	264.67	239.05	507.86	280.30	191.31	379.58
宁夏	310.02	320.45	262.72	250.05	518.83	255.28	194.63	361.52
新疆	308.35	310.22	273.85	239.30	498.32	344.44	178.00	364.88

附录 2：数字普惠金融指标体系与指数计算方法

（一）数字普惠金融指标体系

普惠金融指数科学构建的前提是设计一个完整、准确的普惠金融指标体系。我们在数字普惠金融指标体系的构建过程中遵循了以下原则：第一，同时考虑数字金融服务的广度和深度。数字普惠金融指标体系应该是基于数字金融内涵、特征的综合概括，其所包括的每一个指标和每一个维度都应反映数字普惠金融这一总体的某一个视角。而且不仅要考虑到数字金融覆盖的人群和地域，还要考虑到其被使用的深度，只有这样才能真正刻画出数字金融的普惠价值。第二，兼顾纵向和横向可比性。作为一个动态过程，数字普惠金融的发展随着经济社会和金融体系的发展而不断变化，同一地区在不同年份的数字普惠金融状况会有所变化。此外，不同地区在同一年份由于禀赋、经济发展水平与结构、政策和制度的不同，在数字普惠金融表现上也会存在差异，也需要在数字普惠金融指数上得到体现。因此所编制的数字普惠金融指数最好应该可以同时进行横向（地区维度）比较和纵向（时间维度）比较。第三，体现数字金融服务的多层次性和多元化。现有关于普惠金融的相关研究，主要是从传统银行业务角度来考虑的，随着金融服务的不断创新发展，金融服务已呈现出多层次性和多元化发展的特征。因此，对数字普惠金融的全面刻画要求所构建的指标体系中不仅包括银行服务（主要是信贷），还要包括支付、投资、保险、货币基金、信用服务等业态，以求更加全面地刻画数字普惠金融的发展水平。

按照以上所述的指标体系构建原则，在现有文献和国际组织提出的传统普惠金融指标基础上，结合数字金融服务新形势新特征与数据的可得性和可靠性，我们从数字金融覆盖广度、数字金融使用深度和普惠金融数字化程度等3个维度来构建数字普惠金融指标体系。具体而言，目前数字普惠金融指数一共包含上述3个维度，共计33具体指标[⑥]。具体指标如附表11所示，而数字普惠金融指标体系框架则如附图1所示。

在数字金融覆盖广度方面，不同于传统金融机构触达用户的直接体现为“金融机构网点数”和“金融服务人员数”，在基于互联网的数字金融模式下，由于互联网天然不受地域限制，数字金融服务供给在多大程度上能保证用户得到相应

[⑥] 在第一期指数（2011-2015 年）中共包含 26 个指标。

服务是通过电子账户数体现的。此外，根据金融监管部门的规定，第三方支付的账户如果不绑定银行卡，就只具备小额转账的功能，其价值将大大受限。因此绑定银行卡的第三方支付账户，才是真正有效的第三方支付账户，即实现了对这个用户真正的覆盖。特别是随着第三方支付功能的越来越丰富，第三方支付已经成为重要的理财、融资通道，因此绑定的银行卡数量越多，其理财、转账的覆盖面就越广，对这个账户所有人的金融服务覆盖面就越广，因此一个账户绑定多少银行卡数量也成为数字金融覆盖广度的一个子指标。

在数字金融使用深度方面，本文主要从实际使用数字金融服务的情况来衡量。就金融服务类型而言，则包括支付服务、货币基金服务、信贷服务、保险服务、投资服务和信用服务。从使用情况来看，既包括实际使用总量指标（每万支付宝用户数中使用这些服务的人数），也包括使用活跃度指标（人均交易笔数、人均交易金额）。

在普惠金融数字化程度方面，便利性、低成本和信用化等都是影响用户使用数字金融服务的主要因素，这切实体现了数字金融服务的低成本和低门槛优势，因此普惠金融数字化程度也成为数字普惠金融指标体系的重要组成部分。具体而言，数字金融服务越便利（例如移动支付笔数占总支付笔数的比例高）、成本越低（例如消费贷和小微企业贷利率低）、信用化程度越高（例如免押金支付笔数占总支付笔数比例高），则意味着数字普惠金融的价值就得到更好的体现。

附表 11 数字普惠金融指标体系

一级维度	二级维度		具体指标
覆盖广度	账户覆盖率		每万人拥有支付宝账号数量
			支付宝绑卡用户比例
			平均每个支付宝账号绑定银行卡数
使用深度	支付业务		人均支付笔数
			人均支付金额
			高频度（年活跃 50 次及以上）活跃用户数占年活跃 1 次及以上比
	货币基金业务		人均购买余额宝笔数
			人均购买余额宝金额
			每万支付宝用户购买余额宝的人数
	信贷业务	个人消费贷	每万支付宝成年用户中有互联网消费贷的用户数
			人均贷款笔数
			人均贷款金额
		小微经营者	每万支付宝成年用户中有互联网小微经营贷的用户数
			小微经营者户均贷款笔数
			小微经营者平均贷款金额

	保险业务	每万支付宝用户中被保险用户数
		人均保险笔数
		人均保险金额
	投资业务	每万人支付宝用户中参与互联网投资理财人数
		人均投资笔数
		人均投资金额
	信用业务	自然人信用人均调用次数
		每万支付宝用户中使用基于信用的服务用户数（包括金融、住宿、出行、社交等）
数字化程度	移动化	移动支付笔数占比
		移动支付金额占比
	实惠化	小微经营者平均贷款利率
		个人平均贷款利率
	信用化	花呗支付笔数占比
		花呗支付金额占比
		芝麻信用免押笔数占比（较全部需要押金情形）
		芝麻信用免押金额占比（较全部需要押金情形）
	便利化	用户二维码支付的笔数占比
		用户二维码支付的金额占比

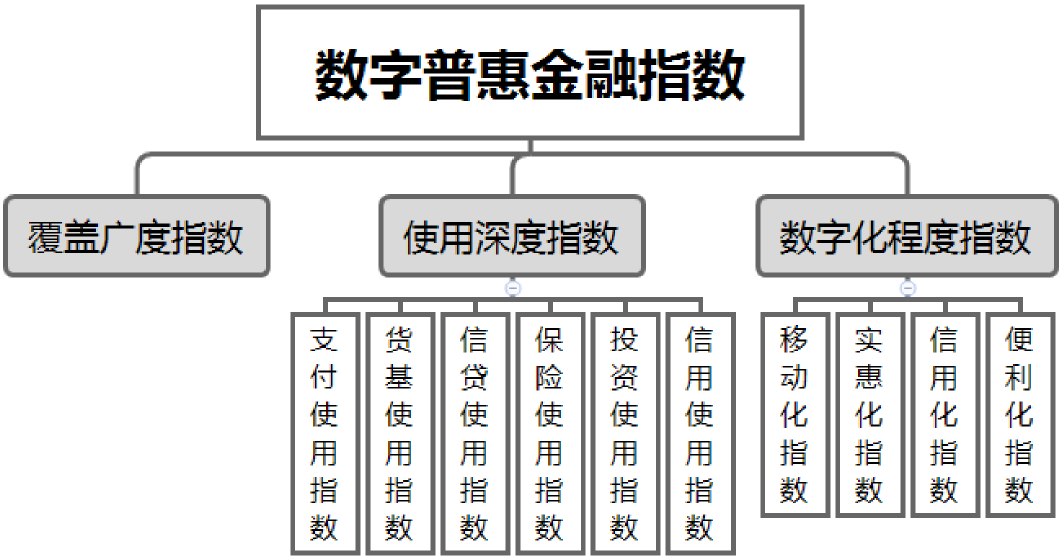

附图 1 数字普惠金融指数框架

（二）指标无量纲化方法

数字普惠金融不同维度的指标虽然都包含了数字普惠金融某些方面的有用信息，但如果单独使用某一个指标或者某一维度指标，又可能会导致对数字普惠金融现状的片面解读，因此，可以参考传统普惠金融指数编制的方法，将数字普惠金融的多个指标合成一个数字普惠金融指数。不少机构和学者都在编制普惠金融指数方面进行了诸多努力和尝试，为我们提供了非常好的借鉴（Sarma，2012；王伟等，2011；伍旭川和肖翔，2014；焦瑾璞等，2015；陈银娥等，2015）。

在指数合成之前，首先必须先将性质和计量单位不同的指标进行无量纲化处理。无量纲化函数的选取，一般要求严格单调、取值区间明确、结果直观、意义明确、不受指标正向或逆向形式的影响。现有文献一般都采用功效函数的方法。在功效函数多指标综合评价体系中，常见的功效函数很多，有线性功效函数法（或称传统功效函数法）、指数型功效函数法、对数型功效函数法、幂函数型功效函数法等等（彭非等，2007）。目前学术界关于普惠金融指标的无量纲化方法主要有线性功效函数法和指数型功效函数法（Sama，2012；焦瑾璞等，2015；伍旭川和肖翔，2014）。本文结合数字金融快速扩张的特点，为缓解极端值的影响，保持指数的平稳性，采取对数型功效函数法。具体而言，对数功效函数的公式如下：

$$d = \frac{\log x - \log x^l}{\log x^h - \log x^l} \times 100 \qquad (1)$$

关于功效函数公式中阈值的确定，如果取各指标不同年份的最大值、最小值作为上下限，当最大值或最小值为极端值或异常值时，容易扭曲指数值，导致地区指数异常。另外，如果各指标的上下限都是基于每年指标情况来设定，会导致不同年份各地区间的指标比较基准发生变化，从而纵向不可比。因此，为了便于今后对各地区数字普惠金融发展水平同时进行横向和纵向比较，我们作了如下处理：（i）对于正向指标，取固定2011年各地区指标数据实际值的95%分位数为上限 x^h，5%分位数为下限 x^l；（ii）对于逆向指标，取固定2011年各地区指标数据实际值的5%分位数为 x^h，95%分位数为 x^l。此外，为了平滑指数，避免种种原因导致的极端值的出现，我们对超过指标上限的地区进行“缩尾”处理：如当某地区基准年（2011年）的指标值超过该指标的上限 x^h 时，则令该地区2011年指标值为上限值 x^h，当某地区2011年的指标值小于其下限 x^l 时，则令该地区2011年指标值为其下限值 x^l。

根据上述方法，我们就可以计算出某年某地区某指标无量纲化后的数值，在基准年（省级和地市级基准年为2011年，县域则以2014年为基准年），每个相应指标的无量纲化数值得分区间在0和100之间，得分越高的地区，相应指标的发展水平就越高。基准年之后年份的数据，指标的功效分值有可能小于0或大于100。

（三）层次分析法

指标无量纲化之后的任务就是确定不同指标合成时的权重。确定权重的方法有很多，根据计算权重时原始数据的来源不同，大体上可分为主观赋权法和客观赋权法两大类。主观赋权法主要由专家根据经验主观判断而得到，如Delphi法，层次分析法（Analytic Hierarchy Process）等；客观赋权法主要是依据各指标的具体数值计算而得到，它不依赖于人的主观判断，因此客观性较强，但不能反映决策者的主观要求，常见方法包括主成分分析法、变异系数法等。主观赋权法和客观赋权法各有优劣，本文采用了主观赋权与客观赋权相结合的方法来确定权重。具体而言，先利用变异系数法求各具体指标对上一层准则层的权重，再通过层次分析法求各准则层指标对上层目标的权重，最后求得总指数。

变异系数法定权重的基本思路是根据各个指标在所有评价对象上观测值的变异程度大小，对其进行赋权，如果一项指标的变异系数较大，那么说明这个指标在衡量该对象的差异上具有较大的解释力，则这个指标就应该赋予较大的权重。变异系数法的具体步骤这里就不展开了。而层次分析法是一种系统分析与决策的综合评价方法，它较合理地解决了定性问题定量化的处理过程。层次分析法的主要特点是通过建立递阶层次结构，把人们的判断转化为若干因素两两之间的重要性比较，从而把难于量化的定性判断转化为可操作的定量判断。“数字普惠金融体系”层面下的三个维度，我们认为数字金融覆盖广度是前提条件，使用深度代表实际使用情况，而数字化程度可以被看作是潜在条件。这里，前两者是“普”的体现，后者是“惠”的体现。因此，根据这三者的相对重要性，我们构建了如附表12所示的判断矩阵。

附表 12 数字普惠金融体系判断矩阵

	覆盖广度	使用深度	数字化程度
覆盖广度	1	2	3
使用深度	1/2	1	2
数字化程度	1/3	1/2	1

对于“使用深度”层面下的6个金融业务维度，我们则按照金融服务的门槛（复杂性和风险性）和普及程度作为判断标准，普及程度越高门槛越低的业务权重越低，相反则越高。据此，从而得到以下附表13的判断矩阵：

附表 13 使用深度判断矩阵

	支付	货基	信用	保险	投资	信贷
支付	1	1/2	1/3	1/4	1/5	1/6
货基	2	1	1/2	1/3	1/4	1/5

信用	3	2	1	1/2	1/3	1/4
保险	4	3	2	1	1/2	1/3
投资	5	4	3	2	1	1/2
信贷	6	5	4	3	2	1

对于“数字化程度”层面下的4个维度，我们按照其对实际生活影响的重要性和业务成熟程度作为判断标准，业务越不成熟、对实际生活影响越小的业务权重越低，相反则越高。据此，得到以下附表14的判断矩阵：

附表 14 数字化程度判断矩阵

	信用化	便利化	实惠化	移动化
信用化	1	1/2	1/3	1/4
便利化	2	1	1/2	1/3
实惠化	3	2	1	1/2
移动化	4	3	2	1

最后，在运用判断矩阵确定各指标权重时，实际上是构造判断矩阵的特征向量。通过解正互反矩阵的最大特征值，可求得相应的特征向量，经归一化后即为权重向量。最后将通过一致性检验的判断矩阵最大特征值所对应的特征向量进行归一化即可得到该层各因素对上层因素的权重大小。三个判断矩阵对应的权重向量如附表14、附表15和附表16所示。

附表 14 数字普惠金融体系下三个维度权重向量

维度	覆盖广度	使用深度	数字化程度
权重	54.0%	29.7%	16.3%

附表 15 使用深度下六个业务维度权重向量

业务维度	支付	货基	信用	保险	投资	信贷
权重	4.3%	6.4%	10.0%	16.0%	25.0%	38.3%

附表 16 数字化程度下四个指标的权重向量[7]

业务维度	信用化	便利化	实惠化	移动化
权重	9.5%	16.0%	24.8%	49.7%

（四）指数合成方法

在完成指标无量纲化处理和确定指标权重后，就可以进行指数合成了。可用于合成的数学方法很多，常见的合成模型有加权算术平均合成模型、加权几何平均合成模型，或者加权算术平均和加权几何平均联合使用的混合合成模型。在综合比较了三种合成方法之后，我们选用了算术平均合成模型，其中主要考虑到该

[7] 这里需要特别说明的是，数字化程度当中“实惠化”、“移动化”两项的权重经过了人工干预，重新分配以保持之前 2011-2015 期指数 1：2 的权重比例，维持指数的延续性。

方法的如下特点：在根据对数功效函数计算各指标得分时，各年都以2011年相应指标值的上下限为比较基准，因此指标无量纲得分有可能为0或负数，为避免最终加权汇总指数值为0，应采取算术加权平均法。加权算术平均合成模型的公式如下：

$$d = \sum_{i=1}^{n} w_i d_i \qquad (2)$$

其中，d 为综合指数，w_i 为各评价指标归一化后的权重，d_i 为单个指标的评价得分，n 为评价指标的个数。

具体指数合成时，是由下往上逐层汇总而成，先计算各层分组指数，然后由各层分组指数加权汇总得到综合指数。其中，在计算数字金融的使用深度指数时，由于六块金融业务开始产生的时间不一致，需逐步纳入指数中，为保证指数的稳定性，此时通过权重归一化使得各块业务之间的相对权重保持一致。如2012年只有支付、信贷和保险三项业务，三项业务的权重分别为：

支付权重=4%/(4%+16%+38%)=7.3%

保险权重=16%/(4%+16%+38%)=27.3%

信贷权重=38%/(4%+16%+38%)=65.4%

当 2013 年开始有互联网货币基金业务时，则相对权重就调整为支付、信贷、保险和货基四项业务权重归一化。其他新出现的业务依此类推。通过逐层算数加权平均合成模型即可计算出最终的数字普惠金融指数。

附录 3：使用数字普惠金融指数的代表性学术成果

[1] 郭峰、王靖一、王芳、孔涛、张勋、程志云，《测度中国数字普惠金融发展：指数编制与空间特征》，《经济学季刊》，2020 年第 19 卷第 4 期，第 1401-1418 页。

[2] 傅秋子、黄益平，《数字金融对农村金融需求的异质性影响：来自数字普惠金融指数与中国家庭金融调查的证据》，《金融研究》，2018 年第 11 期，第 68-84 页。

[3] 何宗樾、宋旭光，《数字金融发展如何影响居民消费》，《财贸经济》，2020 年第 8 期，第 65-79 页。

[4] 何宗樾、张勋、万广华，《数字金融、数字鸿沟与多维贫困》，《统计研究》，2020 年第 10 期，第 79-89 页。

[5] 侯世英、宋良荣，《数字金融对地方政府债务融资的影响》，《财政研究》，2020 年第 9 期，第 52-64 页。

[6] 刘俊杰、李超伟、韩思敏、张龙耀，《农村电商发展与农户数字信贷行为——来自江苏“淘宝村”的微观证据》，《中国农村经济》，2020 年第 11 期，第 97-112 页。

[7] 唐松、伍旭川、祝佳、《数字金融与企业技术创新——结构特征、机制识别与金融监管下的效应差异》，《管理世界》，2020 年第 5 期，第 52-66 页。

[8] 钱海章、陶云清、曹松威、曹雨阳，《中国数字金融发展与经济增长的理论与实证》，《数量经济技术经济研究》，2020 年第 6 期，第 26-46 页。

[9] 邱晗、黄益平、纪洋，《金融科技对银行行为的影响：基于互联网理财的视角》《金融研究》，2018 年第 11 期，第 17-30 页。

[10] 孙玉环、张汀昱、王雪妮、李丹阳，《中国数字普惠金融发展的现状、问题及前景》，《数量经济技术经济研究》，2021 年第 2 期，第 43-59 页。

[11] 万佳彧、周勤、肖义，《数字金融、融资约束与企业创新》，《经济评论》，2020 年第 1 期，第 71-83 页。

[12] 完颜瑞云、锁凌燕，《保险科技对保险业的影响研究》，《保险研究》，2019 年第 10 期，第 35-46 页。

[13] 王靖一，《现金贷果如洪水猛兽？来自断点回归的证据》，《金融研究》，2018 年第 11 期，153-171 页。

[14] 王修华、赵亚雄，《数字金融发展是否存在马太效应?——贫困户与非贫困户的经验比较》，《金融研究》，2020 年第 7 期，第 114-133 页。

[15] 王瑶佩、郭峰，《区域数字金融发展与农户数字金融参与:渠道机制与异质性》，《金融经济学研究》，2019 年第 2 期，第 84-95 页。

[16] 王诗卉、谢绚丽，《经济压力还是社会压力：数字金融发展与商业银行数字化创新》，《经济学家》，2021 年第 1 期，第 100-108 页。

[17] 吴雨、李成顺、李晓、弋代春，《数字金融发展对传统私人借贷市场的影响及机制研究》，《管理世界》，2020 年第 10 期，第 53-64 页。

[18] 谢绚丽、沈艳、张浩星、郭峰，《数字金融能促进创业吗：来自中国的证据》，《经济学季刊》，2018 年第 17 卷第 4 期，第 1557-1580 页。

[19] 易行健、周利，《数字普惠金融发展是否显著影响了居民消费——来自中国家庭的微观证据》，《金融研究》，2018 年第 11 期，第 47-67 页。

[20] 张勋、万广华、张佳佳、何宗樾，《数字经济、普惠金融与包容性增长》，《经济研究》，2019 年第 8 期，第 71-86 页。

[21] 张勋、杨桐、汪晨、万广华，《数字金融发展与居民消费增长：理论与中国实践》，《管理世界》，2020 年第 11 期，第 48-63 页。

[22] 赵涛、张智、梁上坤，《数字经济、创业活跃度与高质量发展——来自中国城市的经验证据》，《管理世界》，2020 年第 10 期，第 65-76 页。

[23] 周利、冯大威、易行健，《数字普惠金融与城乡收入差距："数字红利"还是"数字鸿沟"》，《经济学家》，2020 年第 5 期，第 99-108 页。

附录 4：北京大学数字金融研究中心简介

北京大学数字金融研究中心（Institute of Digital Finance, Peking University）于 2015 年 10 月经北京大学校长办公会批准成立。中心致力于开展对数字金融、普惠金融、金融改革等领域的学术、政策、行业研究，向社会公众提供权威的研究分析，为行业发展提供专业的理论指导，为政府决策提供科学的政策参考。中心目前已有 28 名全职、兼职研究人员。

自成立以来，中心取得了丰硕的研究成果。中心的研究品牌包括北京大学数字金融系列指数与“北京大学数字金融研究中心新金融书系”。北京大学数字金融系列指数由中心研究人员独立或联合开发、发布，分别是《北京大学互联网金融发展指数》、《北京大学数字普惠金融指数》、《北京大学金融科技情绪指数》和《北京大学商业银行互联网转型指数》。北京大学数字金融研究中心新金融书系是中心打造的兼具理论、实践、政策价值的权威性品牌，目前已经出版了《互联网金融 12 讲》、《数字普惠金融的中国实践》、《金融科技的中国时代：数字金融 12 讲》、《蚂蚁金服：从支付宝到新金融生态圈》、《科技赋能：中国数字金融的商业实践》、《数字金融的力量：为实体经济赋能》等著作。此外，中心开展了包括：数字金融支持实体经济发展、数字技术推动普惠金融实践、个人征信体系建设、商业银行应对互联网金融转型策略、个体网络借贷平台的风险、大数据金融等多项研究课题。中心每年还联合经济学和金融学权威期刊举办学术年会，增进数字金融领域内的学术交流，2016 年、2017 年和 2018 年分别与《China Economic Journal》、《经济学（季刊）》和《金融研究》合作。

中心定期举办政策研讨会、北大数字金融讲坛等活动，为学界、商界、政府决策部门提供交流平台。每年 11 月举办的包括来自“一行两会”监管部门领导、高校与研究机构研究人员以及行业从业专家共同参加的中心年会，是数字金融领域的最具影响力的活动之一。中心还在北京大学开设数字金融讲座课，讲授数字金融的前沿问题。

作为重要的智库机构，中心研究人员以大量企业调研数据为基础，在互联网金融与系统性风险、网络借贷平台监管和支持央行推动 G20 框架下以数字金融推动普惠金融发展等议程中积极建言献策，多项成果得到了决策层领导的关注和批示。2018 年，中心联合 24 家中国高校院系和科研机构共同发起中国数字金融研

究联盟，旨在加强中国学术界关于数字金融/金融科技/互联网金融/金融大数据分析等领域的学术研究和人才培养方面的交流合作。

此外，中心还开展了多项国际交流活动，包括与上海新金融研究院联合组织赴欧美发达或新兴经济体考察金融科技行业发展现状和路径，与上海新金融研究院、IMF组织年度国际会议，与日内瓦研究院和诺丁汉大学等国际机构联合召开国际学术研讨会等。
