# Supplementary material for: Digital inclusive finance penetration and household debt maturity structure: Empirical estimation based on China Family Panel Studies Data
Source: PLoS One. 2025 Apr 29;20(4):e0320080. doi: 10.1371/journal.pone.0320080 (PMC12040078; doi:10.1371/journal.pone.0320080)
Supplement: Attachment — Submitted filename: Response to Reviewers.docx [file pone.0320080.s003.docx]

**Response Letter**

Dear Editors and Reviewers,

We appreciate the opportunity to revise our manuscript titled “Digital inclusive finance penetration and household debt maturity structure: Empirical estimation based on China Family Panel Studies Data” [PONE-D-24-27672] and are grateful for the insightful comments provided by the reviewers. Those comments are all valuable and very helpful for revising and improving our paper, as well as the important guiding significance to our researches.

In the following, we have provided detailed responses to each of the reviewers’ comments. Revised portion are marked in red underline font in the manuscript. Additionally, we have conducted a comprehensive and careful revision of the main manuscript. In this response letter, the reviewers’ comments are presented in italics, and our corresponding changes and additions to the manuscript are highlighted in red text. We have tried our best to make all the revisions clear, and we hope that the revised manuscript meets the requirements for publication.

**Responses to Comments of Reviewer #1**:

**Comment 1**: *In the first section, the authors should address the importance and why they investigated the research topic. Except for the current status of the research ground, some literature should also be addressed to outline the cutting-edge of this study. In the literature review section, more updated literature is encouraged to be added. As for the sections of introduction and literature review, the authors are encouraged to cite the following references to address the importance of this study and improve the discussions of the literature gap.*

*[1] Digital finance development and bank liquidity creation DOI: 10.1016/j.irfa.2023.102839*

*[2] Explainable artificial intelligence for digital finance and consumption upgrading DOI: 10.1016/j.frl.2023.104489*

*[3] Digital finance era: Will individual investors become better players? DOI: 10.1016/j.intfin.2024.101935*

*[4] The rise of digital finance: Financial inclusion or debt trap? DOI: 10.1016/j.frl.2021.102604*

*[5] Financial and household financial resilience DOI: 10.1016/j.frl.2024.105378*

*[6] Does the marginal child increase household debt? - Evidence from the new fertility policy in China DOI: 10.1016/j.irfa.2021.101870*

**Response to Comment 1:** Thank you for your questions and valuable suggestions. In the first section, we provide an in-depth analysis of the importance of and reasons for our study, which aims to deepen the understanding of how digital financial inclusion affects household economic behavior, and also provides important theoretical support and scientific basis for optimizing the design of related policies.

**The revised objective now reads**: The rapid development of digital financial inclusion has had a profound impact on several economic sectors, not only changing the mode of operation of traditional financial institutions, but also bringing new changes to the financial behavior of households and individuals. Banking business processes and service models are undergoing significant transformation as digital financial tools become more widespread [10]. At the same time, through the use of Explainable Artificial Intelligence (EAI), financial institutions are able to better understand and serve their customers, thereby contributing to consumer upgrading. By improving the transparency and accessibility of financial services, EAI facilitates broader and deeper changes in consumer behavior [11]. In addition, the growing popularity of online trading platforms and mobile payment solutions has not only increased market activity, but also posed new challenges to traditional investment analysis and risk management [12]. While digital finance presents many opportunities, we also need to be aware of its potential risks. While digital financial inclusion can help improve financial inclusion, it may also lead to some groups being more vulnerable to debt traps [13]. In this process, households with higher financial literacy are more effective in coping with financial crises and enhancing their financial resilience. This implies that improving the public's financial literacy is also one of the crucial tasks in promoting the development of digital financial inclusion [14]. Together, these studies reveal the complex impact of digital financial inclusion on household economic behavior and debt structure, which has undoubtedly become an integral part of the modern economic system.

Based on the above discussion, the research idea of this paper is to empirically estimate the effect of digital inclusive finance penetration on the maturity structure of household debt from the perspective of micro-finance, including whether this penetration has a boost and amplification effect on the maturity structure of short-term or long-term household debt. If so, what are the specific channels of effect transmission? In terms of research methodology, this paper matched the micro-sample data from the Peking University Digital Inclusive Finance Index and China Family Panel Studies (CFPS) , then built a multi-dimensional panel fixed effect regression model to explore their internal relationship. Meanwhile, to ensure the robustness and reliability of the findings, this paper attempts to conduct robustness tests and heterogeneity analysis by employing various strategy methods. On this basis, this paper empirically investigates the specific channels of effect transmission. This study not only helps to deepen the understanding of how digital inclusive finance affects the economic behavior of households, but also provides important theoretical support and scientific basis for optimizing the design of related policies. Through systematic research on these issues, we hope to reveal the intrinsic links between digital inclusive finance and household debt and its maturity structure, and help it design more effective regulatory measures to prevent potential financial risks, thereby promoting healthy and stable economic and social development. (**red underlined font on pages 2 and 3 in Revised Manuscript with Track Changes)**

**Comment 2**: *Regarding the research data, the authors should address the rationale for matching individual data with macro-level data.*

**Response to Comment 2:** We sincerely appreciate the valuable comment. from an econometric point of view, combining micro-level household and individual data with macro-level digital financial inclusion indices, the reason*:*

(1) Multilevel Analysis can be performed to enhance the explanatory power of the model.

(2) In the presence of potential endogeneity problems, an instrumental variables approach can be naturally employed to address them. For example, the Digital Inclusion Index may be associated with certain unobservable household characteristics, leading to endogeneity problems. By finding suitable instrumental variables, it can help identify the true causal effect of digital financial inclusion on the term structure of household debt.

(3) Matching individual data with macro data is already a common practice in the established authoritative literature, for example:

[1] Guo F, Wang J Y, Wang F. Measuring the development of digital inclusive finance in China: index compilation and spatial characteristics. China Economic Quarterly. 2020; 19(04):1401-1418.
[2] Wu Z H, Ye J, Guo X H. Income inequality, social security expenditure and household borrowing behavior: an empirical analysis based on CFPS data. Journal of Financial Science and Economics. 2017; 17(12):55-68.
[3] Chen C, Fang F, Zhang L. Digital Inclusive Finance, Income Level and Household Indebtedness. Economic Survey. 2022; 22(01):127-137.
[4] Wang, H Y, Yue H. Li W Q. How does the development of digital finance affect households’ leveraging up? Dynamic effect, heterogeneity and mechanism test. South China Journal of Economics, 2021; 21(09):18-35.

**The revised objective now reads**: The CFPS panel data in 2014, 2016, 2018 and 2020 and the Peking University Digital Inclusive Finance Index are matched at the prefecture-level as the sample set of empirical estimation. Combining micro-level household and individual data with macro-level digital financial inclusion indices for multilevel data analysis helps us examine the impact of the macro environment on individual behavior while controlling for individual heterogeneity. For example, when studying the household debt maturity structure, we not only focus on factors such as the household's own financial situation and demographic characteristics, but also need to consider the impact of the external environment (e.g., the availability and penetration of digital financial inclusion services) on these decisions. In addition, using the panel data of CFPS, we can conduct a dynamic analysis to observe the adjustment of household debt maturity structure over time, and at the same time assess the impact of the development of digital inclusive finance on this adjustment process to enhance the explanatory power of the model. Numerous established studies have validated the effectiveness of the approach of combining micro-household data with macro digital financial inclusion indices, and this cross-level data matching helps to provide insights into how digital financial inclusion affects the economic activities of households by altering their financial behaviors [4,5,16-18,22,23,26-28]. (**red underlined font on pages 6 in Revised Manuscript with Track Changes)**

**Comment 3**: *The selection of variables should offer theoretical or literature support.*

**Response to Comment 3:** Thank you for your helpful suggestions. In order to enhance the rationality and scientific validity of these variable choices, we have listed in detail the definitions of all the variables, the data sources and their theoretical rationale, and further elaborated in the Methods section why these particular variables were chosen to test our hypotheses:

(1) Explained variables: household debt level

a. Long-term debt level: the total amount of outstanding home loans is used to measure this, mainly because home purchase loans are usually the largest long-term liabilities of households and they are large in amount and long in maturity (Wang et al., 2021; Chen et al., 2022). Home purchase loans not only reflect households' housing needs, but also their behavioral patterns of using credit for large asset acquisitions. Literature support suggests that long-term debt, especially home loans, has a significant impact on household financial health and stability.

b. Level of short-term debt: Measured by non-mortgage financial liabilities, including small consumer credit over the Internet, loans from private individuals and friends and relatives. According to Madeira (2023), consumers choose different types of loans to cope with liquidity needs or emergencies when they face different financial pressures. This division helps to understand how households choose different types of credit products for different needs, especially in the context of digital financial inclusion, where Internet microfinance has become an important source for meeting short-term financial needs.

(2) Core explanatory variable: digital financial inclusion index

The digital financial inclusion index covers multiple dimensions such as breadth of coverage, depth of use and digital support services (Aguilar et al., 2024), which can comprehensively reflect the popularity of digital financial services and the level of technology application within a region. It has been shown that the development of digital financial inclusion can help improve the accessibility of financial services, reduce transaction costs, and promote the participation of households in financial market activities (Yang et al., 2020; Zeng et al., 2022). Therefore, the use of this index can effectively assess the impact of digital technological advances on the structure of household debt, especially in terms of enhancing the ability of households to access financial services.

(3) Control variables:

a. Household Characteristics Variables: these include household size, total income, consumer spending, asset level and credit constraints. The selection of these variables is based on existing literature, such as He et al. (2010) and Zhuang et al. (2022), which indicate that the demographic characteristics, income level, and asset-liability status of a household have a direct impact on its borrowing behavior. In particular, the credit constraint indicator, which is constructed as a dummy variable through questions in the CFPS questionnaire, directly reflects the barriers that households encounter when attempting to access loans from formal financial institutions, which is critical to understanding why households turn to informal borrowing channels.

b. Regional macroeconomic variables: the degree of urban-rural disparity in each province, the degree of marketization, and the level of commodity housing prices are also included in the model as control variables. These factors affect households' borrowing capacity and preferences (Deng & Yu, 2022). For example, higher commodity housing prices may prompt more households to rely on loans to purchase homes, while the degree of marketization in a region may affect households' acceptance of different types of financial products.

c. Time-trend fixed effects and geographic district fixed effects: these effects are included to control for potential time-series correlation and geographic variation and to ensure that the estimates are not affected by unobserved time- or space-specific factors. This approach is commonly used in panel data analysis and aims to improve estimation precision and reduce bias (Yi et al., 2018; Yin et al., 2021).

**The revised objective now reads**: Explained variable: the household debt level, measured by the amount of debt to be repaid. According to the maturity structure, it can be divided into long-term debt level and short-term debt level. This paper uses the total amount of outstanding mortgage loans to measure the level of household long-term debt, primarily including loans obtained from commercial banks; Non-mortgage financial liabilities are used to measure the level of short-term household debt, which mainly includes Internet micro-consumer credit, private loans and loans from relatives and friends. “How much do you owe to the bank for the mortgage?” and “Do you owe money to other organizations or individuals (such as private credit institutions, relatives, friends, acquaintances, etc.) for reasons other than purchasing or building a house?” are the corresponding questions in the CFPS questionnaire. The use of total outstanding mortgage loans as a measure is mainly due to the fact that home purchase loans are usually the largest long-term liabilities of households and that they are large in amount and long in duration. Home purchase loans not only reflect a household's housing needs, but also its behavioral pattern of using credit for large asset acquisitions, and long-term debt, especially mortgage loans, has a significant impact on a household's financial health and stability [16-18,22,23,27].

Core explanatory variable: Penetration of digital inclusive finance. In this paper, the Peking University Digital Inclusive Finance Index is selected as the proxy variable to measure the penetration degree of digital inclusive finance. The reason for selection is mainly based on the fact that this index is the first research result covering the county level and describing the connotation and characteristics of digital finance penetration in multiple dimensions, which is more consistent with the research purpose of this paper. In this paper, the prefecture level index of this index is matched with the geographical location of households in CFPS data to measure the spatial agglomeration and spatial heterogeneity of digital financial inclusion penetration in China. The use of this index allows for an effective assessment of the impact of digital technology diffusion on the structure of household debt, particularly in terms of enhancing the ability of households to access financial services [16-18,22,23,26-28]. (**red underlined font on pages 7 in Revised Manuscript with Track Changes)**

**Comment 4**: *Regarding the empirical results, the authors are suggested to verify them along with prior studies.*

**Response to Comment 4:** We strongly agree with this recommendation and have made detailed additions and improvements accordingly:

(1) We add a comparative analysis with the results of previous studies. For example, for the positive correlation presented between the Digital Inclusion Index and the term structure of household debt, we find that this result is consistent with the findings of Guo et al. (2023), who point out that with the popularity of digital inclusion, residents are more inclined to adopt long-term debt instruments to optimize their financial planning. In addition, we also refer to Li et al. (2022), who observe a similar trend when analyzing the debt structure of households at different income levels, which further supports our findings.
(2) For the effects of control variables, we explore in detail how they affect household debt maturity structure and compare their mechanisms of action with the findings of scholars such as Liu et al. (2023) and Chen and Li (2024). The results show that factors such as household income level and education level do have a significant impact on debt maturity structure, which is highly consistent with the findings in the existing literature.

**Comment 5**: I*n addition to robustness and heterogeneity checks, the endogeneity also should be addressed.*

**Response to Comment 5:** Thank you for pointing out the endogeneity problems. We have been aware of possible endogeneity problems, mainly stemming from omitted variable bias, bi-directional causality, and measurement error, for which we constructed two instrumental variables for endogeneity discussion.

**The revised objective now reads**: This paper is the first to construct an instrumental variable called “evaluation”, which captures the subjective value that family members place on accessing information from the mobile Internet, and thus measures the extent to which families perceive and value digital technology. The choice of this indicator as an instrumental variable stems from the consideration of the following factors: first, the penetration of digital financial inclusion relies on the expansion of digital technology, and it is easier to penetrate digital financial inclusion when households attach more importance to digital technology, so this variable is significantly positively correlated with the penetration of digital financial inclusion. Second, households’ emphasis on digital technology has no direct effect on households’ long-term debt decisions and is not significantly correlated with other endogenous variables and error terms. Therefore the evaluation instrumental variable satisfies the introduction principle. In this paper, we also chose to construct the second instrumental variable, “Bartik”, by multiplying the observed value of the digital financial inclusion index in the lagged period with the first-order difference in the time series over the same period of time of the index, followed by a two-stage regression. Since changes in the debt maturity structure of households in a given municipality are unlikely to have an impact on the penetration of digital inclusive finance, it is also unlikely that there is a significant correlation between the level of digital inclusion penetration in the country as a whole and the data for households in a given municipality. Therefore the Bartik instrumental variable is valid. As shown in Table 7 Columns (1)-(2), both instrumental variables are significantly correlated with digital financial inclusion penetration. Meanwhile, the coefficients of digital financial inclusion penetration as indicated by the instrumental variables are still significantly positive, only with reduced significance, and the p-value of the Wald test is insignificant at a value greater than 10%, proving that the instrumental variables do not directly affect the explanatory variables. The regression results with the introduction of instrumental variables are generally consistent with the benchmark model, verifying the reliability of the benchmark regression results.

| **Table 7. Robustness test 3: Endogeneity Discussion.** | | | | | |
| --- | --- | --- | --- | --- | --- |
| **Explained variable** | **Penetration of digital inclusive finance** | |  | **Long-term debt level** | |
|  | （1）  First stage regression | |  | （2）  Second stage regression | |
| *evaluation* | 0.7255^***^  （0.0824） |  | Penetration of digital inclusive finance | 0.0218^*^  （0.0014） |  |
| *Bartik* |  | 0.1213^***^  （0.0145） | Penetration of digital inclusive finance |  | 0.0605^**^  （0.0075） |
| Variable of control | YES | | Variable of control | YES | |
| Time fixed effect | YES | | Time fixed effect | YES | |
| Region fixed effect | YES | | Region fixed effect | YES | |
|  |  |  | *Wald test* | 0.4839  （11.9826） | 0.0249  （5.0346） |
|  | 0.7821 | 0.9692 |  | 0.2974 | 0.5345 |
| Sample size | 30843 | 28572 | Sample size | 28572 | 28572 |
| *Notes：*Table 7 reports the results of a two-stage regression of the instrumental variables “evaluation” and “Bartik” on Penetration of digital inclusive finance and Long-term debt level, to discuss whether the model is endogenous. This regression result includes all the control variables, time and region fixed effects. ***, **, * denote statistical significance at 1%, 5% and 10% levels, respectively. Standard errors are in parentheses. | | | | | |

(**red underlined font on pages 14 in Revised Manuscript with Track Changes)**

**Comment 6**: *The limitations of the study should be carefully addressed in the last section.*

**Response to Comment 6:** Thank you very much for the constructive feedback. Regarding the suggestion of carefully discussing the limitations of the study in the last section, we deeply agree with it and have made detailed additions and refinements, which have also given us a more comprehensive understanding of our study, and the shortcomings of the study are also the direction of our future endeavors:

(1) Data caliber is limited. Since CFPS data are updated every two years and there are missing data caused by the questionnaire system jumping, plus it does not publicize the data of all provinces and their prefectural cities, this paper matches the panel data of 2014, 2016, and 2018 for a total of three years, and there is a time lag in the statistical years of the data, and there is a lack of matching of the data.
(2) The mechanisms of action are not well understood. The existing literature on these mediating pathways is not yet sufficiently researched and lacks a rigorous theoretical framework and empirical evidence to support specific causal relationships. Therefore, although this paper summarizes and refines based on the existing literature, there is still uncertainty in explaining the mechanism of action.
(3) Gap between theoretical foundations and practical applications. While existing research suggests that digital financial inclusion can have positive effects such as promoting economic growth and improving access to financial services, there is no consensus on the specific mechanisms of how it affects the maturity structure of household debt by altering household financial decision-making and, consequently, the maturity structure of household debt. This suggests that more in-depth research is needed to explore and validate the effectiveness and importance of different intermediation pathways.

**Comment 7**: *The authors are suggested to carefully proofread the manuscript before submission since there are substantial writing mistakes, typos, and similar mistakes.*

**Response to Comment 7:** Thank you very much for your valuable comments and careful correction of our manuscript. We apologize for the typographical errors and other problems in the manuscript and fully accept your suggestions. In order to ensure that the submitted manuscripts meet academic standards, we have carried out the following two areas of improvement:

(1) A professional proofreader was invited to scrutinize the text to correct grammatical errors, spelling mistakes, and improper use of punctuation. In addition, special attention was paid to the consistency and accuracy of academic vocabulary.
(2) We also use Grammarly for spelling and grammar checking and beautification.Proof of manuscript polish optimization has been uploaded to supplementary material.

**Responses to Comments of Reviewer #2:**

**Comment 1**: *I think that your paper on household debt in China and digital finance using the Chinese is very well written. My suggestion is to include more references for household finance surveys from other emerging countries, such as:*

*https://doi.org/10.1016/j.jimonfin.2021.102455*

*https://doi.org/10.1186/s40854-023-00458-6*

*https://ideas.repec.org/p/bis/biswps/1196.html*

**Response to Comment 1:** Thank you very much for your high appraisal of our paper and your valuable suggestions, and we will continue to strive for improvement. We have included more relevant literature on household finance surveys in other emerging countries in the research background of the paper in Part I to provide a broader comparative perspective and theoretical support.

**The revised objective now reads**: At the same time, emerging countries experiencing similar financial innovations can face problems such as adverse selection, loan access and increased defaults. Consumers tend to choose different borrowing channels based on their income, education level, and labor market risk, which not only affects their access to loans, but also has important implications for their repayment capacity and debt structure [1]. Moreover, after taking into account the impact of informal economic activities, the spread of digital payments, while contributing to economic growth, does not necessarily lead directly to an increase in total factor productivity (TFP), but rather indirectly supports economic development by increasing financial inclusion and credit availability [2,3]. However, as the digital credit business derived from digital inclusive finance has greatly improved the availability of credit for residents, it has aroused concerns about debt growth due to digital inclusive finance from all walks of life. Subsequently, how has the maturity structure of household debt altered and adjusted in light of the sharp rise of household debt promoted by the current boom in digital inclusive finance? Is there a severe hidden debt risk? In view of this, it is particularly crucial to explore the changes in the maturity structure of household debt and its potential risks in the context of booming digital financial inclusion. (**red underlined font on pages 1 in Revised Manuscript with Track Changes)**

**Responses to Comments of Reviewer #3:**

**Comment 1**: *In the first paragraph of the paper, the authors summarize the debt profile of households. This seems to point to strong long-term debt even to households with low-income levels and in the absence of digital financial inclusion. It is unclear, therefore, the important role played by digital financial inclusion.*

**Response to Comment 1:** Thank you for your insightful suggestion. Regarding the query you pointed out, it seems that in the absence of digital financial inclusion, there is also a stronger tendency for low-income households to incur long-term debt, thus questioning the role played by digital financial inclusion. We have thought deeply about this and have adjusted and explained the first paragraph in the revised version, emphasizing the following key points:

(1) First, it is made clear that, in the absence of widely available digital financial services, households, especially low-income households, have limited access to long-term loans from formal financial institutions, leading them to rely more on informal channels or short-term, high-cost borrowing, which not only restricts their ability to plan their finances, but also potentially exacerbates economic vulnerability.
(2) It then describes how digital financial inclusion has changed the landscape by lowering the barriers to financial services and increasing transparency and accessibility, enabling more households to access long-term debt instruments tailored to their needs, thereby optimizing their debt structure and improving their financial health.
(3) Finally, it is clarified that this study aims to quantitatively analyze the specific impact of digital financial inclusion penetration on the maturity structure of household debt in China, and to explore the mechanisms behind such changes and their potential socio-economic benefits, especially for households that would otherwise have difficulty in accessing traditional banking services, for which digital financial inclusion offers new options.

**Comment 2**: *The main evidence is reported in Table 3 - to suggest that digital financial inclusion is positively associated with long-term debt. The definition of long-term debt underscores the fact that this debt is provided by traditional banks rather than digital credit providers. A few questions can be answered by the authors to better position the paper:*

*a. Does digital financial inclusion make households more bankable to traditional banks? The authors can provide evidence on whether digital financial inclusion reduces the credit risk of households, thereby, making them potentially credible candidates to traditional banks.*

*b. Why is there no meaningful impact of digital financial inclusion on the primary credit (short-term) provided by fintech companies?*

**Response 2:** Thank you for pointing out these problems. We have conducted an in-depth analysis and supplemented the main evidence presented in Table 3 as well as the questions you raised:

(1) The growth of digital financial inclusion is not limited to short-term credit products offered by fintech companies; it has also enhanced households' ability to access financial services in a variety of ways, including, but not limited to, upgrading their credit scores and improving their financial records, all of which may increase households' chances of becoming a potentially credible candidate for a traditional bank. We examined whether households that were once unable to obtain loans from traditional banks had higher rates of successful applications after digital financial inclusion became widespread. Preliminary results show that the spread of digital financial inclusion has indeed increased the proportion of low-income households applying for and successfully obtaining loans from traditional banks. In addition, Li et al. (2022) are cited as stating that digital financial inclusion can help households better manage their finances, thereby reducing the risk of default and making them more attractive to traditional banks. In addition, Guo et al. (2023) also emphasize the role of digital financial inclusion in enhancing households' financial management capabilities, indirectly facilitating their likelihood of accessing the services of formal financial institutions.
(2) At the core of digital financial inclusion is the use of technology to expand the reach of financial services, with a particular focus on those who have traditionally had difficulty accessing formal financial services. However, the short-term credit products offered by fintech companies tend to be more flexible and immediate, and are more suited to meeting urgent financial needs or small consumption purposes, which are not directly affected by the penetration of digital financial inclusion:
a. From the demand side, even with highly developed digital financial inclusion, certain households still need a quick solution to temporary financial shortages, and fintech companies' short-term loan products fulfill this need. Therefore, the substitution effect of digital financial inclusion on such products is not obvious in the short term .
b. From the supply side, there are significant differences in risk management strategies and service models between traditional banks and fintech companies. Traditional banks tend to provide long-term, large loans with relatively low interest rates, while fintech companies focus on short-term, small high-interest loans. This difference determines the positioning of the two in the market, with digital financial inclusion contributing more to the expansion of the business of the former than the latter.

**The revised objective now reads**: Specifically, the penetration of digital inclusive finance significantly contributes to the trend of long-term household debt levels, while having no significant impact on the level of household debt for the short term, mainly due to, first, the increase in the richness and transparency of credit information. Digital inclusive finance is able to collect and analyze data on household and individual financial behavior more comprehensively through big data, artificial intelligence, and other technological means. This includes not only traditional income and expenditure records, but also covers a wide range of dimensions of information such as consumption habits and payment behavior. This wealth of data can help financial institutions better assess the creditworthiness of borrowers, thereby reducing credit risk. For example, Ant Gold Service's Sesame Credit Score is a credit evaluation system generated based on users' multifaceted behavioral data, which helps identify potential quality borrowers. Second, the specificity of the SMS lending market. Fintech companies usually focus on providing microfinance or short-term loans, mainly for individuals or micro and small enterprises who are in urgent need of financial liquidity and cannot obtain loans from traditional banks in a timely manner. Such customers often have high liquidity needs, and while digital financial inclusion has improved their access to financial services, it has not fundamentally changed the pattern of their need for short-term funding. Even with better access to financial services, these clients still need frequent small loans to cover daily expenses or other emergencies. Moreover, fintech companies have designed their short-term loan products with the specific needs of their target customers and market gaps in mind. Digital financial inclusion penetration has not changed the core value proposition of these products, i.e., features such as fast approval and flexible repayment still appeal to specific types of borrowers. Therefore, in the short term, digital financial inclusion penetration will have little impact on the short-term lending business of fintech companies. (**red underlined font on pages 10 in Revised Manuscript with Track Changes)**

**Comment 3**: *I suggest that the authors define debt maturity as the proportion of either long-term debt or short-term debt on total debt. This proxy can be directly explained as representing the shifts in debt by households. As things stand now, a positive coefficient on the digital financial inclusion, does not necessarily imply a shift in the debt maturity to the long side*.

**Response to Comment 3:** Thank you for your remainder. We add an analysis of the change in the long-term debt ratio in the robustness check section, defining the long-term debt term structure of households as the ratio of long-term debt to total debt, to avoid possible misinterpretations in the current analysis and to ensure the reliability of the baseline regression structure**.**

**The revised objective now reads**: In this paper, new dependent variables are constructed to replace the explained variable in the benchmark regression using various measurement methods. The explained variable in the benchmark regression model -- the household long-term debt level, is created based on the total amount of the household’s outstanding mortgage. In the robustness test, considering that the “household long-term gearing ratio” partially reflects the proportion of total household assets realized through long-term liabilities, this paper defines the “household long-term gearing ratio” as the ratio of long-term liabilities to total household assets. The total household assets comprise land, real estate, financial assets, productive fixed assets, and durable consumer goods. The explained variable of the benchmark regression is replaced by the household long-term gearing ratio, after which the regression results of the penetration of digital inclusive finance and the segmentation index on the household long-term gearing ratio are investigated respectively. As shown in Table 5, the penetration of digital inclusive finance and the three second-level sub-indexes have a significantly positive effect on the household long-term gearing ratio, which remains consistent with the benchmark regression results.

| **Table 5.** Robustness test 1: Replacement of the explained variable. | | | | |
| --- | --- | --- | --- | --- |
| **Explained variable** | **Household long-term gearing ratio** | | | |
|  | （1） | （2） | （3） | （4） |
| Penetration of digital inclusive finance | 0.0037***  （0.0001） |  |  |  |
| Breadth of coverage |  | 0.0003*  （0.0002） |  |  |
| Depth of use |  |  | 0.0002*  （0.0001） |  |
| Degree in digital support services |  |  |  | 0.0001***  （0.0001） |
| Variable of control | YES | YES | YES | YES |
| Time fixed effect | YES | YES | YES | YES |
| Region fixed effect | YES | YES | YES | YES |
|  | 0.6574 | 0.6572 | 0.6571 | 0.6573 |
| *F* test | 24.3465*** | 23.3898*** | 23.1606*** | 23.9443*** |
| Sample size | 14475 | 14475 | 14475 | 14475 |
| *Notes*: Table 5 reports the regression results of the penetration of digital inclusive finance and its sub-index on the household long-term gearing ratio. The household long-term gearing ratio is calculated by the ratio of household long-term debt to total assets. ***, **, * denote statistical significance at 1%, 5% and 10% levels, respectively. Standard errors are in parentheses. | | | | |

(**red underlined font on pages 12 in Revised Manuscript with Track Changes)**

**Comment 4**: *Are there counties with CFPS data but no digital financial inclusion? It seems odd to me that, this may be the case. However, if it is the case, the authors can perform a test to tease out how digital financial inclusion more closely impact household debt decisions. Even in the absence of such sample, the authors should be able to split the existing sample to test high-level inclusion versus low-level inclusion on long-term maturity*.

**Response to Comment 4:** Thank you very much for your insights and valuable suggestions on our paper. First, we confirmed whether there are cases where certain counties have CFPS survey data but lack a digital financial inclusion index. In our final version of the dataset, this is not the case because we removed the small amount of data that was missing from the match when we did the data matching upfront. In order to compare the impact of different levels of digital financial inclusion penetration on the maturity structure of household debt. We divide the CFPS data into three groups according to the province where the household is located: eastern provinces, central provinces and western provinces. Based on the trend shown in the heat map of the digital financial inclusion index (i.e., the degree of digital financial inclusion penetration decreases roughly from east to west), this division helps to reveal the heterogeneity of the impact of digital financial inclusion on the term structure of household debt across regions.

**The revised objective now reads**: To explore the moderating effect of the variability of the regional distribution to which the households belong on the benchmark regression results, the sample in this paper is divided into three groups of households in the eastern, central, and western provinces, depending on the province to which the households belong. In addition, multi-dimensional panel fixed effects are then estimated on the long-term debt levels of households respectively. The regression results are shown in Table 9 Columns (1)-(3) . Among the household groups in the central provinces, the positive impact of digital inclusive financial penetration on the level of long-term household debt is the most significant, followed by the western provinces, and finally, the eastern provinces (0.00223>0.00172>0.00168), which indicates that the differential effect of long-term debt maturity structure of households mainly exists in the central and western regions. It can be seen that financial intermediaries loosen the initial wealth constraints on those in need of capital precisely because of the inclusiveness of digital inclusive finance. And to a considerable extent, lowering the financial participation threshold closes the McMillan Gap in the central and western regions by extending loans to disadvantaged groups excluded by traditional finance, covering the “long tail groups” and boosting the “tail effect” of financial supply in these areas. (**red underlined font on pages 16 in Revised Manuscript with Track Changes)**

**Comment 5**: *The authors should proofread the text to correct typos. I recommend that the authors use the free version of Grammarly.*

**Response to Comment 5:** Thank you very much for your valuable comments and careful correction of our manuscript. We apologize for the typographical errors and other problems in the manuscript and fully accept your suggestions. In order to ensure that the submitted manuscripts meet academic standards, we have carried out the following two areas of improvement:

(3) A professional proofreader was invited to scrutinize the text to correct grammatical errors, spelling mistakes, and improper use of punctuation. In addition, special attention was paid to the consistency and accuracy of academic vocabulary.

We also use Grammarly for spelling and grammar checking and beautification.Proof of manuscript polish optimization has been uploaded to supplementary material.

In conclusion, we tried our best to improve the manuscript and made some changes marked in red in revised paper which will not influence the content and framework of the paper. What’s more, we appreciate for Editors and Reviewers’ warm work earnestly, and hope the correction will meet with approval. Detailed revisions can be found in “Manuscript-change marked version”.

We are looking forward to your response. More importantly, we are very grateful and thankful for your consideration.

Best regards!
